# Effectiveness of Physical Activity Interventions on Pregnancy-Related Outcomes among Pregnant Women: A Systematic Review

**DOI:** 10.3390/ijerph16101840

**Published:** 2019-05-23

**Authors:** Carmen W. H. Chan, Elce Au Yeung, Bernard M. H. Law

**Affiliations:** The Nethersole School of Nursing, The Chinese University of Hong Kong, Hong Kong, China; whchan@cuhk.edu.hk (C.W.H.C.); bernardlaw@cuhk.edu.hk (B.M.H.L.)

**Keywords:** physical activity, intervention, pregnant women

## Abstract

Regular physical activity has been demonstrated to contribute to physical and psychological health. Nevertheless, pregnant women generally exhibit low levels of physical activity. Implementation of interventions that enhance the self-efficacy of pregnant women on increasing physical activity is required. This paper provides an in-depth review of studies reporting the effect of various physical activity interventions dedicated for pregnant women on pregnancy-related issues, including gestational weight gain, pain and depression, physical activity level, and quality of life among these individuals. Five databases were used in searching the literature. Findings of the included studies were presented narratively, and appraisal of their methodological quality was conducted using the quality assessment tool developed by Effective Public Health Practice Project. Review findings demonstrated that physical activity interventions are effective in enhancing physical activity levels of pregnant women. Further, they are potentially useful in alleviating pregnancy-related pain and psychological symptoms, reducing gestational weight gain, and increasing self-efficacy in enhancing physical activity levels among these individuals. Nevertheless, inconsistencies in findings between studies hamper the drawing of firm conclusions on these latter outcomes. Overall, studies demonstrated a positive effect of physical activity interventions on the well-being and physical and psychological health of pregnant women.

## 1. Introduction

Regular physical activity is known to be beneficial to both physical and psychological health [1]. The World Health Organization recommends that adults aged between 18–64 should engage in at least 150 min of moderate-intensity activity or 75 min of vigorous-intensity physical activity per week [2]. Lack of adequate physical activity was identified as the fourth leading cause for global mortality attributed to cardiovascular disease, stroke, and diabetes mellitus [3]. Nevertheless, a local survey showed that only 50.7% male and 37.4% female adults have achieved a higher level of physical activity recommended by the WHO [4]. Likewise, a study involving pooled analysis of population-based surveys on physical activity levels also revealed a rather stable prevalence of low physical activity levels among populations worldwide over the past 15 years [5]. In view of the lack of a decreasing trend in the prevalence of insufficient physical activity among populations worldwide, strategies need to be developed to effectively educate individuals on the importance of physical exercise. Among these individuals, pregnant women are in high need of such education. 

### 1.1. Physical Inactivity among Pregnant Women

Pregnant women are likely to exhibit sedentary behaviors and be physically inactive, owing to their need to adjust to considerable physiological and psychological changes during pregnancy [6]. Indeed, a previous study in the United States showed that only 32% of pregnant women exhibited physical activity levels that were able to meet the physical activity guidelines during early pregnancy, while this number was reduced to 12% by late pregnancy [7]. Likewise, a study with Asian subjects also revealed that the total energy expenditure of women during their pregnancy was significantly lower than that before they became pregnant [8]. Consistent with this, a previous review has also reported the consistent finding that physical activity level progressively decreases among pregnant women throughout their pregnancy [9]. Moreover, pregnant women were reported to spend over 50% of their time without physical activity [10]. Previous studies have also compared the proportion of pregnant women being adequately active physically with that of nonpregnant women. Results showed that a considerably lower proportion of pregnant women appeared to exhibit a physical activity level that met the recommended guidelines, compared to nonpregnant counterparts (15.8% vs. 26.1%) [11]. Further, pregnant women were also found to spend significantly more time exhibiting sedentary behavior compared to nonpregnant women [12]. All these data indicate a reduction in physical activity level among pregnant women and suggest the potential existence of barriers for these women to perform sufficient exercise during their pregnancy. 

Previous studies have also explored the factors that hamper the maintenance of a sufficient physical activity level among pregnant women. Indeed, pregnant women were reported to often experience a repertoire of pregnancy-related symptoms, including lumbopelvic pain [13], psychological issues, such as anxiety and depression [14], and gestational weight gain [15], some of which would likely present a barrier for pregnant women to exhibit a higher level of physical activity [16,17]. Some pregnant women also indicated that a lack of time available for physical exercises, due to their busy work schedule, was also a contributing factor in their lack of physical activity [18]. Moreover, pregnant women may also possess misconceptions on the effect of physical exercises on fetal health. For example, many pregnant women expressed concerns on the potential harm of physical activity to the fetus [18,19]. In particular, Chinese pregnant women were reported to be reluctant to engage in prenatal physical activity and perceive it as an antenatal taboo, as they believe that physical activity during pregnancy may pose potential harm to the fetus and could result in miscarriage [19,20]. Consistent with these findings, many studies worldwide have reported a decrease in duration, intensity, and/or frequency of physical activity among women since the prepregnancy period [8,11,12,21,22,23,24,25]. In light of the above data on the low physical activity levels among pregnant women and the barriers they face in enhancing their physical activity levels, pregnant women appear to be in need of receiving physical activity interventions in order to increase their awareness on the benefits of physical activity on their health and well-being. Indeed, it was previously suggested that pregnancy is one of the periods where women can be effectively motivated to modify their health behavior, including their physical activity habits [26].

### 1.2. Benefits of Physical Activity during Pregnancy

Findings from a number of systematic reviews have demonstrated the benefits of physical activity during pregnancy on the physical health of pregnant women. Through a meta-analysis of twelve randomized controlled trials, Streuling et al. demonstrated that pregnant women subjected to physical activity interventions are less likely to exhibit excessive gestational weight gain compared to controls [27]. Physically active pregnant women were also shown to have a lowered risk of gestational diabetes mellitus [28,29], although there is a current lack of sufficient evidence to support the effectiveness of physical activity interventions in preventing gestational diabetes mellitus [30,31]. Two systematic reviews showed that physical exercises during early pregnancy may protect women from developing preeclampsia [32,33]. A recent systematic review also demonstrated the benefits of exercises on the reduction of the severity of low back pain and lumbopelvic pain [34]. Of note, while it was shown that both land-based and water-based exercises would exhibit beneficial effects on pregnant women, it was previously shown that exposure to disinfectants used in pools for water-based activities could contribute to detrimental effects on fetal growth [35], thereby raising concerns as to the appropriateness of water-based exercises among pregnant women. 

Moreover, physical activity during pregnancy was also shown to confer psychological health to pregnant women. Physically active pregnant women are more likely to perceive themselves to achieve a better health status than sedentary women [36], and they have a lower risk of developing perinatal depression [37,38,39]. Notably, engagement in moderate-intensity exercise from early to late pregnancy is not associated with preterm delivery and it does not affect the birth weight of the baby [7,40,41]. The promotion of physical activity, through the implementation of physical activity interventions dedicated for pregnant women, should therefore be encouraged.

In light of the benefits of physical activity for pregnant women, various countries have published guidelines on the recommended level of physical activity that pregnant women should attain. Nevertheless, there are variations in the recommended duration and frequency of exercise in the guidelines imposed by different countries, as indicated in a previous review [42]. For example, while guidelines published by the United States suggest the performance of moderate-intensity exercise for 30 min for most days of the week, the Japanese guidelines recommend a duration of 60 min of aerobic exercise for 2–3 times per week, and the Norwegian guidelines suggest aerobic exercise for 30 min every day per week. Overall, worldwide guidelines recommend a total of 120–210 min of physical activity per week for pregnant women.

### 1.3. Intervention on Prenatal Physical Activities

To date, only a limited number of studies reported the development of prenatal physical activity interventions that are based on theoretical frameworks such as Social cognitive theory only [43]; Social cognitive theory and the Transtheoretical model [44,45], and Protection motivation theory [46]. Overall, use of theoretical frameworks might allow better explanation of which components are effective on the outcomes of intervention and facilitate the generalization of results to a particular population. Nevertheless, Currie et al. (2013) reported in a systematic review the effectiveness of behavioral change interventions on augmenting the level of physical activity among pregnant women in eight out of ten included studies [47]. Individualized goal planning was commonly employed in these studies for intervention development and these educational interventions yielded positive outcomes among pregnant women. However, interventions reported by only two of the included studies that showed desirable outcomes [48,49] were delivered by nurses, while most of these included studies were delivered by individuals of various disciplines, including nutritionists, physiotherapists, or exercise specialists [50,51,52]. 

Although reviews have previously been published summarizing the previously developed physical activity interventions for pregnant women and assessed their effects on the aforementioned pregnancy-related symptoms through meta-analyses, few reviews have set out the major components of these interventions (defined as the strategies used in the interventions to enhance physical activity levels among pregnant women), and the theoretical frameworks used in their development, which may provide clues on how these interventions may have exhibited their effectiveness. A systematic review of physical activity interventions dedicated to pregnant women and their effectiveness in (1) addressing pregnancy-related symptoms, (2) increasing exercise intentions, and (3) improving quality of life among these women would be required, in order to provide clues to the future development of appropriate and effective physical activity interventions for pregnant women on promoting physical activity. The review also serves to identify major and effective components of these interventions. 

### 1.4. Objectives

The objectives of this review are to provide an in-depth review on the major components of physical activity interventions involving land-based exercises that are dedicated for pregnant women and to examine the effectiveness of the interventions on improving exercise self-efficacy, physical activity levels, and pregnancy-related outcomes including depression, pregnancy-related pain, and gestational weight gain.

## 2. Methods

### 2.1. Search Strategy

Literature search was conducted in September 2018 using five databases including PubMed, OVID MEDLINE, EMBASE, PsycINFO, and CINAHL. The search strategy, depicting the combination of keywords used for the literature search, is presented in Table 1.

The selection of search terms used in the search strategy was based on the objectives of the review, where the effect of interventions, including web-based interventions and those led by nurses, with a physical activity or exercise component dedicated for pregnant women on a variety of outcomes was examined. These outcomes include women’s intention or self-efficacy in doing physical exercises, physical activity level, pregnancy-related outcomes, including weight issues and psychological issues, and parameters that would affect quality of life, such as pain, sleep difficulties, and functional impairments.

### 2.2. Inclusion and Exclusion Criteria

Included studies are original articles reporting randomized controlled trials (RCT) or controlled clinical trials (CCT). These trials should report the effectiveness of physical activity interventions or lifestyle interventions that contain a physical activity component on one or more of the following outcomes: (1) exercise self-efficacy, (2) physical activity levels, (3) depression, (4) lumbopelvic pain, and/or (5) gestational weight gain, among pregnant women. The included studies should involve intervention participants who are healthy pregnant women aged 18 or above, who carry a singleton pregnancy and should be free from medical or obstetrical contraindication against physical activity in pregnancy. All studies published before September 2018 that fit the above criteria are included in this review. 

Studies involving pregnant teenage women are excluded as these women are likely to be underweight in early pregnancy, and are more likely than adults to gain an excessive weight throughout pregnancy [53]. Inclusion of studies involving teenage pregnant women in this review could therefore result in misleading interpretation of intervention effects on gestational weight gain. Moreover, articles that are not published in English and those that report study protocols, case reports, or qualitative studies are also excluded. 

### 2.3. Data Extraction and Summary

Selection of studies to be included in this review was first conducted by one author through the screening of the title and abstract of the retrieved articles, based on the inclusion and exclusion criteria presented above. The results of article selection were then verified and confirmed by a second author, who acts as the second reviewer. Articles with abstracts that appear to fit the inclusion criteria were subjected to further screening through reading their full text to confirm their eligibility for inclusion.

Data extraction was first performed by one author, and the extracted data were then verified by a second author. Extracted data include study design, study settings, characteristics of study participants, sample size, deliverer of the interventions, major physical activity components of the interventions, outcome measures of interest, data collection time points, outcome assessment tools used, models used as theoretical framework for the development of interventions, and major findings of the studies. Any disagreements in the extracted data between the two authors were resolved by discussion to reach a consensus.

Owing to the diversity in the nature of the interventions and the outcome measures involved in the included studies, it was not possible to conduct a meta-analysis on the extracted data. The extracted data were therefore presented in a narrative and tabular manner. Significance of the differences in the outcome measures reported in the included studies were indicated using *p* values.

### 2.4. Critical Appraisal on Methodological Quality of Included Studies

The methodological quality of the controlled trials reported in the included studies was assessed using “The Quality Assessment Tool for Quantitative Studies” developed by the Effective Public Health Practice Project (EPHPP), a tool for assessing the methodological quality of public health studies [54]. This tool has previously been used for quality assessment of included studies in systematic reviews on the topic of public health [55]. In the assessment, the studies were rated as either strong, moderate, or weak for each of the following six categories: controlling for selection bias, study design, controlling for confounders, blinding, data collection methods, and withdrawals and dropouts. The category “selection bias” assesses whether the recruited sample was likely to be representative of the target population and how large the proportion of the approached potential participants that have agreed to participate in the intervention is. The category “study design” evaluates whether the study is of a randomized controlled design, and whether the randomization methodology is sound. The category “confounders” assesses whether the baseline characteristics of the participants in the intervention and control groups are similar, and whether confounders are controlled. The category “blinding” assesses whether the outcome assessors are blinded to group allocation and whether study participants are aware of the outcomes assessed. The category “data collection methods” evaluates the suitability of the data collection instruments used, such as their validity and reliability. The category “withdrawals and dropout” assesses the extent of participant withdrawals in the reported intervention, which may affect the interpretation of intervention effectiveness. The global methodological quality rating for each included study was assigned based on the ratings for each category as instructed [54]. A study was considered to have a “strong’ methodological quality rating if no weak ratings were assigned to any of the above categories. If a study received a weak rating to one of the categories, it was then considered to have a “moderate” methodological quality rating. “Weak” methodological quality rating was given to a study if it received “weak” ratings in two or more categories. The appraisal was performed by one author, and the ratings were independently verified by a second author.

## 3. Results

### 3.1. Search Results

**Figure 1:**
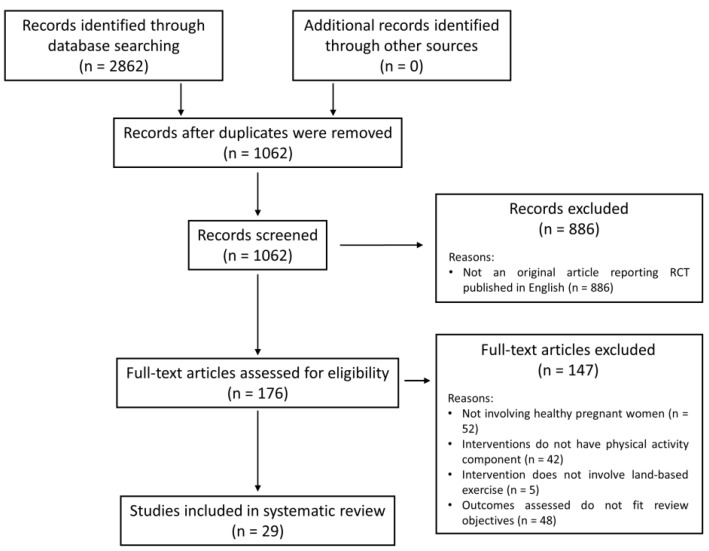
Flow of literature search and article selection.

A total of 2862 citations were retrieved using the search strategy presented in Table 1. After the removal of duplicates (*n* = 1800), the remaining 1062 citations were screened for eligibility for inclusion. Citations that are not original articles reporting RCT or CCT and not published in English (*n* = 886) were removed. One hundred and seventy-six citations were further screened for eligibility and 147 citations were further excluded as they do not match the eligibility criteria. A total of 29 articles were included for review. A Preferred Reporting Items for Systematic Reviews and Meta-Analyses (PRISMA) diagram depicting the flow of literature search and article selection is presented in Figure 1.

#### 3.1.1. Methodological Quality of Included Studies

The included studies were rated on their methodological quality based on six broad categories, namely selection bias, study design, confounders, blinding, data collection methods, and withdrawals and dropouts. Overall, these studies were generally rated weak in their methodological quality. The majority (*n* = 17; 58.6%) of the studies were rated moderate for the category of ‘selection bias’, with most (*n* = 22; 75.9%) of the studies determined to have a sample of participants that is somewhat likely to be representative of the target population. About 50% of these studies reported that over 80% of the potential participants approached had agreed to participate in the reported interventions, although nine of them (31.0%) did not report the total number of potential participants approached during subject recruitment. The majority (*n* = 25; 86.2%) of the studies were rated strong for the category of ‘study design’, in which the reported methods used for randomization were considered appropriate. Likewise, most (*n* = 27; 93.1%) of the studies were rated strong for the category of ‘confounders’, where these studies reported nonsignificant differences in the baseline characteristics of the participants from the intervention and control groups. In addition, the majority (*n* = 23; 79.3%) of the included studies were also rated strong for the ‘data collection method’ category, with the instruments used for outcome assessment in these studies having been shown to be valid and reliable. Nevertheless, a significant proportion (*n* = 16; 55.2%) of the included studies were rated weak for the ‘blinding’ category, as there was no evidence supporting that the outcome assessors and the participants in these studies were not aware of the outcome of group allocation and the research question of the studies, respectively. Finally, the vast majority of the included studies were rated either strong (*n* = 14; 48.3%) or moderate (*n* = 12; 41.4%) for the category of ‘withdrawals and dropouts’, with at least 60% of the enrolled participants involved in the studies being able to complete the reported interventions. A summary of the methodological quality ratings of the included studies is presented in Table 2.

#### 3.1.2. Characteristics of Included Studies

Table 3, Table 4, Table 5, Table 6 and Table 7 presents the characteristics of the included studies. The publication dates of these studies range between 2000 and 2018. Most of them were conducted in Europe (*n* = 16; 55%), of which nine were in Norway [56,57,58,59,60,61,62,63,64], two in Turkey [65,66], two in Spain [39,67], and one each in Croatia [68], Finland [69], and Sweden [70]. Seven studies (24%) were conducted in the Americas, with two in Brazil [71,72], two in Canada [52,73], two in Columbia [74,75], and one in the United States [76]. Further, two studies were conducted in Iran [77,78], two were conducted in Taiwan [48,79], and one each in South Africa [80] and Thailand [81]. There was a large variation in the sample size of the studies, ranging from 20 to 962. The majority of the included studies were RCTs, except one which was a controlled clinical trial [69]. Four of these studies were of a secondary analysis of a previously conducted trial [61,63,64,68].

The physical activity interventions reported by the included studies were either conducted in local hospitals [39,56,57,58,65,68,71,74,75,77,81], healthcare or medical centers [48,59,66,67,79], clinics [60,61,69,70,78,80], or local community [52,62,63,64,73,76], although one study involved the conduction of the intervention in both hospitals and clinics [72]. These interventions were either led by nurses [48,65,69], midwives [70,77], physiotherapists [56,57,58,59,71], aerobic or exercise instructors [52,62,63,64,73,76,81], fitness specialists/exercise professionals [39,72], or the investigators of the study [66,79,80]. Nevertheless, interventions reported by four studies were led by physiotherapists coupled with either students at the fitness centers [60,61] or physicians [74,75]. Additionally, three studies did not specify who was involved in the delivery of the intervention [67,68,78]. 

In order to measure the outcomes for the assessment of intervention effects, the majority of the included studies utilized validated instruments and tools, although some conducted the one or more outcome measurements using either author-developed questionnaires [56,59,61,63], weight measurements [60,62,66,67,69,72,76], logbooks [52,73], or medical records [39,73,78]. However, one study did not report the methodologies used in outcome measurements [70].

#### 3.1.3. Effects of Physical Activity Interventions on Pregnancy-Related Outcomes

(1) Pain

Ten of the included studies reported the effect of physical activity interventions on the following two measures on pain: pain intensity and prevalence of pain experience within the sample of participants. While studies consistently reported the lack of a significant effect of the intervention on the number of participants reporting the experience of lumbopelvic pain [56,59,63,68,71], they generally yielded inconsistent findings for the effect of intervention on pain intensity. For example, significant between-group differences (*p* ≤ 0.017) were observed postintervention on the intensity of low back pain [65,77,80,81], lumbopelvic pain [68], and childbirth pain [79]. However, other studies failed to show a significant effect of the reported intervention on such parameters [56,59,71]. Therefore, it is difficult to draw a firm conclusion as to the effect of physical activity interventions on alleviating pain among pregnant women, although they appeared to have no effect on prevalence of pain among intervention participants. However, all studies showing a significant effect on pain intensity demonstrated a significant reduction of pain levels among intervention participants at postintervention, thereby suggesting a positive effect of the reported interventions on alleviation of pregnancy-related pain, including low back pain and lumbopelvic pain. Table 3 summarizes the studies that investigate the effect of physical activity interventions on pregnancy-related pain.

(2) Gestational Weight Gain

Fourteen of the included studies reported the effect of physical activity interventions on maternal or gestational weight gain. Overall, eight of these studies reported no significant effect of the reported interventions on this parameter [52,66,69,72,73,76,77,78]. However, five of them were able to observe a significantly lower maternal/gestational weight gain among the intervention participants after the intervention (*p* ≤ 0.043) [39,48,60,67,70]. Interestingly, Haakstad and Bo reported variations in their findings when different approaches were used in data analysis [62]. Using an intention-to-treat approach, they reported no significant differences (*p* = 0.31) were observed on the level of maternal weight gain between groups. In contrast, this parameter was shown to be significantly lower (*p* = 0.01) among the intervention participants when data analysis was performed in a per-protocol manner, by only including participants who exhibited 100% adherence to the intervention protocol in the analysis. With the variations of findings between these studies, firm conclusion can neither be drawn for the effect of physical activity interventions on reducing weight gain among pregnant women, but they point towards a positive effect of the interventions on the reduction of weight gain. Table 4 summarizes the studies that assess the effect of physical activity interventions on gestational weight gain.

(3) Psychological Outcomes

Seven of the included studies investigated the effect of physical activity interventions on psychological outcomes such as anxiety and depression. Among these studies, inconsistent results were reported in terms of the effect of the reported interventions on the perceived severity of anxiety and depression by the participants. While three of these studies reported no significant difference in anxiety and/or depression levels between groups at postintervention [57,58,71], three other studies were able to show a significantly lower level of depression among the intervention participants after the intervention (*p* ≤ 0.005) [39,48,75]. Further, Haakstad et al. also demonstrated the variations in their findings with respect to the effect of their intervention on the frequency of negative mood feelings experienced by the participants, when different approaches of data analysis were performed [64]. While no significant between-group difference (*p* = 0.4) was observed for this parameter when the intention-to-treat approach was used, a significantly lower number of intervention participants (*p* = 0.01) was found to express negative mood feelings when data were analyzed in a per-protocol manner. Overall, studies reported a generally positive effect of physical activity interventions on addressing psychological issues, such as anxiety and depression, among pregnant women, although some of the included studies failed to show any significant effect on this outcome. Table 5 summarizes the studies that investigate the effect of physical activity interventions on various psychological outcome parameters.

(4) Quality of Life

Among the included studies, only two reported the effect of physical activity interventions on quality of life. While Montoya Arizabaleta et al. showed that their reported intervention could lead to a significantly higher increase in health-related quality of life among the intervention participants compared to controls [74], Haakstad et al. did not observe any significant effect of their intervention on this outcome parameter [64]. With only two studies reporting contrasting effect of physical activity interventions on quality of life of pregnant women, the effectiveness of such interventions on improving the quality of life of these individuals remains elusive. Table 6 summarizes the studies that investigate the effect of physical activity interventions on quality of life.

(5) Physical Activity Level or Self-Efficacy in Physical Activity

Nine studies reported the effect of physical activity interventions on physical activity levels exhibited by pregnant women, or their self-efficacy in increasing physical activity levels. The majority (*n* = 6; 66.7%) of these studies reported a significantly higher level of physical activity among the intervention participants at postintervention (*p* ≤ 0.027) [52,60,68,71,73], or a significantly larger increase in this parameter among intervention participants compared to control counterparts (*p* = 0.0002) [66]. Notably, Miquelutti et al. were able to demonstrate a contrasting effect of their intervention on participants in different groups, with increasing physical activity levels among intervention participants and decreasing physical activity levels among controls after the intervention [71]. Further, Huang et al. demonstrated a significantly greater increase in participants’ self-efficacy in physical activity among the intervention participants compared to controls [48], suggesting the effectiveness of their intervention in encouraging pregnant women to engage in physical exercises. However, Kinnunen et al. failed to observe a significant effect of their intervention in increasing physical activity levels among the participants [69]. In terms of self-efficacy in physical activity, Haakstad et al. also reported a nonsignificant difference in the extent of the decrease in the number of perceived barriers to physical activity between groups [61]. Nevertheless, the effect of physical activity interventions on physical activity levels among pregnant women is consistent overall between the included studies, with most studies demonstrating a positive effect of the interventions on this parameter. More data are required to draw firmer conclusions about the effect of such interventions on self-efficacy in the enhancement of physical activity levels among pregnant women, however. Table 7 summarizes the studies that evaluate the effect of physical activity interventions on participants’ levels of physical activity.

(6) Theoretical Framework

Despite a number of included studies reporting that the exercise interventions were designed based on the recommendations from guidelines proposed by established health-related organizations such as the American College of Obstetricians and Gynecologists, only three of the studies had indicated that the development of the reported interventions were guided by theory-based models. The intervention developed by Aşci and Rathfisch [66] was based on the Pender’s Health Promotion Model, a model that is widely used for the development of interventions that aim to modify poor health behaviors [82]. The model was used for the design of the counselling component of the intervention, where the perceived barriers of the participants to increase in physical activity levels were identified, so that a more effective personalization of the intervention can be achieved. Likewise, Kinnunen et al. [69] utilized the counselling model on physical activity developed by Laitakari and Asikanen [83] for the design of the counselling component of the intervention. This model defines the steps and strategies required to ensure the effectiveness of counselling on physical activity for health promotion, including the exploration of the factors that influence health behaviors. In the Kinnunen et al. study [69], intervention participants were encouraged to discuss on their facilitators and barriers to performing regular exercises, enabling a better understanding by intervention deliverers on the participants’ needs in increasing physical activity levels. In the Ozdemir et al. [65] study, the counselling component of the intervention was delivered according to the principle of adult education, although the authors did not explain how the principle was utilized in guiding the design of the counselling intervention. Nevertheless, owing to the scarcity of studies utilizing a theoretical framework model in guiding intervention development, it is difficult to conclude whether the use of a theoretical framework model would exert benefits in enhancing the effectiveness of physical activity interventions.

(7) Components of the Interventions

##### Intervention Content

Interventions reported among the included studies exhibited a fair degree of variation in their content. However, the majority of these interventions comprised primarily of an exercise class supervised by the intervention deliverer. Some of the interventions additionally include components such as counselling on physical activity via face-to-face sessions [60,65,69,70,71] and dissemination of information and advice on the recommended physical exercise for pregnant women via information booklets, leaflets, and/or websites [56,59,60,65,69,79,80], except two studies which involved counselling sessions and information provision without the use of exercise classes [48,66]. Depending on the objective of the interventions, some interventions include the dissemination of information on pregnancy-related symptoms, such as pain, and their potential management strategies [56,59,65,71,80], aiming to increase the effectiveness of such interventions for the management of pregnancy-related symptoms.

Although the vast majority of the interventions included an exercise class/program for participants to be engaged in, the type of these exercises also exhibited some variation. Such variation can be attributed to the differences in the objectives of the interventions. For example, interventions that address lumbopelvic pain would include exercises that focus on the strength of the pelvic floor muscles. In contrast, interventions that address lifestyle modifications and excessive weight gain would generally include aerobic exercises such as cycling and aerobic dance. Nevertheless, in addition to exercise classes, a number of the reported interventions include a component of home-based exercises, where participants were encouraged to carry out exercises at home on a regular basis according to the instructions provided in the intervention materials [52,56,57,59,63,71,73,78,79,80,81].

Owing to the variations in the findings between studies on the effect of interventions on various outcomes, it is difficult to draw any firm conclusion on whether a particular intervention component is effective in the improvement of an outcome. For example, even though all the reported interventions for addressing low back pain and pelvic girdle pain involve the activation of muscles around the pelvic floor, it is difficult to distinguish whether a specific intervention component is responsible for the effectiveness of these interventions. As presented in Table 3, four studies reported interventions that were effective in addressing low back pain and pelvic girdle pain. However, while some of these effective interventions solely involved the participation in supervised exercise classes, others additionally involved counselling sessions and/or information provision on pain care. It is therefore unclear which of these components are major contributors to the effectiveness of the interventions. Likewise, sole implementation of exercise programs for the participants in the reported interventions was found to produce mixed results between studies, on both gestational weight gain and psychological outcomes, even though similarities in the frequency and duration of these exercise classes were observed between studies. Moreover, very few studies investigating intervention effects on these two parameters involved the inclusion of intervention components other than exercise classes, resulting in difficulties to conclude whether exercise program implementation alone would have any impact on the effectiveness of these interventions in improving gestational weight gain and psychological outcomes.

Nevertheless, as demonstrated in Table 7, it is possible that the delivery of exercise classes to intervention participants could play an important role in enhancing their physical activity levels. Indeed, a generally consistent positive effect of interventions including a compulsory exercise program was reported among studies investigating the effects of interventions on physical activity level. Conversely, the intervention reported by Kinnunen et al. [69] primarily involved counselling on physical activity, while the participation in an exercise class was only optional. Such intervention resulted in nonsignificant effects on participants’ physical activity levels. Of note, several studies investigating the effect of interventions on physical activity levels additionally incorporated strategies for maintaining the healthy behaviors of participants, such as regular telephone reminders by or meetings with the intervention deliverer, encouraging the participants to do exercise at home regularly [66,71,80]. These interventions were shown to exhibit a significant positive effect on participant outcomes, including an increase in physical activity levels. These data suggest that exercise classes, coupled with strategies to encourage health behaviors, could effectively help enhance the physical activity levels of pregnant women.

##### Intervention Dosage

The duration of the reported interventions ranged from four weeks to thirty weeks, while the majority of the interventions lasted for twelve weeks. For interventions that involved the delivery of an exercise program, the majority involved a duration of exercise for 30–60 min at a frequency of 2–3 times per week involving moderate-intensity physical activities such as aerobic exercises. Such exercise dosage is in line with the recommendations stipulated by the American College of Obstetricians & Gynaecologists [84], where pregnant women should engage in regular moderate-intensity physical activity, for at least 20–30 min per day. Nevertheless, one study involved an intervention with an exercise dosage that is lower than the recommended levels, where the intervention participants were asked to do cycling at home for a duration of only fifteen minutes, thrice per week [78]. This intervention failed to exhibit any effect on reducing the gestational weight gain among the participants, thus suggesting a need for effective physical activity interventions to prescribe exercise programs that meet the guidelines on the recommended physical activity levels for pregnant women.

## 4. Discussion

Pregnant women are known to experience pregnancy-related symptoms such as weight gain, anxiety, and depression, with the latter two symptoms being associated with body image dissatisfaction [85]. In light of this, strategies need to be developed to alleviate such symptoms in order to improve their quality of life. As previous studies indicate a benefit of physical exercises on the improvement of both physical and psychological outcomes, it is tempting to speculate that physical activity interventions specifically designed for pregnant women would be effective in addressing the pregnancy-related symptoms mentioned above. This review serves to provide an overview of the interventional studies that investigate the effectiveness of land-based physical activity interventions on the alleviation and/or improvement of a variety of pregnancy-related symptoms among pregnant women.

One major finding of our review is the positive effect of physical activity interventions on enhancing the physical activity level among pregnant women, and this finding is generally consistent among the included studies reporting such effect. Notably, one of the studies even reported contrasting outcomes between participants in the intervention and control groups at postintervention, with intervention participants exhibiting increased physical activity levels and control participants having reduced level of physical activity [71]. These data therefore suggest the effectiveness of physical activity interventions in encouraging pregnant women to do physical exercise more regularly, which would potentially help improve their physical fitness [86]. These interventions generally consist of components including supervised group exercise classes, and/or the provision of information on the recommended level of physical activity for pregnant women via counselling and information booklets, suggesting that these strategies should be sufficient to encourage an increase in physical activity levels among pregnant women. Indeed, increase in physical activity levels would be of benefit particularly for pregnant women, as it was previously demonstrated that physical activity during pregnancy would confer benefits to the health of the fetus, potentially via the maintenance of the vascular function of the placenta [87]. Likewise, physical activity during pregnancy would also help reduce the risk of the development of depression, both at the antenatal and postpartum stages [88,89], and improve body image satisfaction and self-esteem [90]. Furthermore, previous studies have shown that the implementation of exercise programs among pregnant women would help enhance their cardiorespiratory fitness [91], an indicator suggested to be positively correlated with health-related quality of life [92]. All these highlight the importance of the commitment of pregnant women to regular exercises through the implementation of physical activity programs on both the women’s and fetus health. Of note, however, owing to the scarcity of studies on the effect of such interventions on self-efficacy in increase physical activity levels among pregnant women, it is difficult to determine whether the increase in physical activity level of women at postintervention reported by the included studies was due to their increase in self-efficacy to do more physical exercises. More studies on the effect of physical activity interventions on the self-efficacy of pregnant women in doing regular exercises are therefore required.

Nevertheless, our review revealed rather inconsistent findings among the included studies regarding the effect of physical activity interventions on other physical and psychological outcomes, including pain, gestational weight gain, and depression. While one cannot rule out the possibility of the heterogeneity of the nature of the interventions reported in the included studies as a cause for such inconsistencies in findings, it is possible that the variations in methodologies used in data collection among the studies could also be one of the contributing factors. Indeed, different studies utilized different instruments for assessment of the same outcome (Table 3, Table 4, Table 5, Table 6 and Table 7). For example, while some studies used the visual analogue scale for the assessment of perceived pain levels, some utilized an author-developed questionnaire or KEBK questionnaire for this assessment, and different conclusions were drawn from such studies. Indeed, reliable comparisons of study findings can only be performed when all included studies utilize the same instrument for outcome assessment [93]. Furthermore, variations of the methodology used for data analysis between studies could be one of the factors for inconsistencies of study findings. Indeed, some included studies reported a significant effect of the reported interventions on certain outcomes when data were analyzed in a per-protocol manner, but not when analyzed using an intention-to-treat approach [62,64]. These observations therefore indicate a need for caution in the interpretation of the findings of the included studies regarding the significance of the effect of interventions on various outcomes. Nevertheless, with each study reporting a significant effect of the intervention, indicating a positive effect on each of the outcomes investigated in this review (except quality of life), it is likely that implementation of physical activity interventions would also be beneficial for pregnant women in alleviating pain and depression and reducing gestational weight gain, in addition to the increase in physical activity levels. Further studies on physical activity interventions on the quality of life of pregnant women are needed to generate more data for a firmer conclusion to be drawn regarding their effects on this outcome.

Our review has several limitations that should be acknowledged. First, only RCTs and CCTs were included in this review. Data from studies with other study designs, such as one-group pretest-posttest design, were not considered. As these studies may contribute further data on the significance of the effect of physical activity interventions on the outcomes involved in this review, the exclusion of such studies may have limited the comprehensiveness of this review. Second, the review provides a general overview on the effect of physical activity interventions on the outcomes, rather than that of a specific type of physical activity intervention. Different interventions included in this review possess different characteristics that may affect the significance of the effect of the interventions, such as the nature, frequencies, and duration of the intervention and type of intervention deliverer. For example, some interventions primarily included a group exercise program, while a few additionally included the provision of information and counselling on physical activity recommendations and/or the management of various pregnancy-related symptoms. The heterogeneity of multiple confounding characteristics of the reported interventions among the included studies may have resulted in variations in findings between studies, and hamper the drawing of firm conclusions. Third, the review included studies that report lifestyle interventions containing both dietary and physical activity components. As it is difficult to attribute the positive effects of each intervention component to each outcome examined, the reported effectiveness of the interventions on the outcomes may not necessarily be attributed to the effect of the physical exercises involved in these interventions. Fourth, as indicated in Table 3, Table 4, Table 5, Table 6 and Table 7, differential dropout rates were observed between groups among some of the included studies, which could lead to the existence of bias in the reporting of the extent of the intervention effects. Owing to these review limitations, the interpretation of the findings of this review should be performed with caution.

### Implications for Practice and Research

With the finding that physical activity interventions can effectively increase physical activity levels of pregnant women, an initiative that would confer benefits on their health and well-being, the implementation of such interventions for women throughout their pregnancy is warranted. To this end, community health programs dedicated to pregnant women should consider the incorporation of physical activity interventions as an integral component. These interventions should include multiple elements, including compulsory exercise classes, counselling on physical activity, and the dissemination of information on the exercises suitable for pregnant women. Exercise classes should also involve exercises with duration and intensity that are in line with the recommended guidelines for pregnant women to maintain health. These strategies should be effective in encouraging an enhancement in the physical activity levels of pregnant women. Further, pregnant women should be encouraged to take compulsory maternity leave, allowing more time for them to participate in health programs involving physical activity interventions for more effective promotion of health and well-being throughout the period of their pregnancy.

Currently, there is still a scarcity of data on the effect of physical activity interventions on quality of life of pregnant women, and their effects on various pregnancy-related outcomes (such as pregnancy-related pain and psychological issues) remain inconclusive. As these outcomes are highly related to the well-being of pregnant women, the evidence for a positive effect of physical activity interventions on the well-being of these individuals is still lacking. Future research should be directed towards investigation of the effect of such interventions on these outcome parameters, in order to provide more data for more conclusive evidence.

## 5. Conclusions

The performance of adequate physical exercise is of great importance for pregnant women, as it has been shown to be beneficial to the health of both the mother and the fetus. Implementation of interventions that aim to enhance their physical activity levels is therefore recommended. Our review demonstrates that physical activity interventions, especially those with compulsory exercise classes incorporated as an integral component, are effective in enhancing the level of physical activity among pregnant women, and are possibly useful for the enhancement of self-efficacy in physical activity, reduction of gestational weight gain, and alleviation of pregnancy-related symptoms, such as depression and pregnancy-related pain, among these individuals. These interventions may therefore potentially help address the barriers to increasing physical activity levels among pregnant women. Nevertheless, with the variations of findings between studies owing to the heterogeneity of the reported interventions, firm conclusions cannot be drawn regarding the latter outcomes. Future studies may be directed towards the investigation of the effect of physical activity interventions on both the aforementioned outcomes and the quality of life among pregnant women, contributing further data on whether such interventions can promote the well-being of these individuals via the improvement of pregnancy-related symptoms.

## Figures and Tables

**Table 1:** The search strategy.

‘Pregnant women’ OR ‘Pregnancy’ OR ‘Prenatal’ OR ‘Antenatal’ OR ‘Gestation’ OR ‘Maternal’
**AND**
‘Intervention’ OR ‘Program’ OR ‘Program’ OR ‘Therapy’ OR ‘Education’ OR ‘Web-based’ OR ‘E-health’
**AND**
‘Physical activity’ OR ‘Exercise’ OR ‘Land-base exercise’ OR ‘Nurse-led’
**AND**
‘Weight gain’ OR ‘Weight control’ OR ‘Self-efficacy’ OR ‘Depression’ OR ‘Psychological’ OR ‘Pain’ OR ‘Sleep disturbance’ OR ‘Sleep difficulties’ OR ‘Functional ability’ OR Functional status’ OR ‘Sick leave’

**Table 2:** The methodological quality of the included studies.

Author/Year	Methodological Quality Rating (EPHPP)						
	Selection Bias	Study Design	Confounders	Blinding	Data Collection Method	Withdrawals and Dropouts	Overall
Kinnunen et al. 2007	Moderate	Strong	Strong	Weak	Strong	Moderate	**Moderate**
Huang et al. 2011	Moderate	Strong	Strong	Moderate	Strong	Moderate	**Moderate**
Ozdemir et al. 2015	Moderate	Strong	Strong	Weak	Strong	Strong	**Moderate**
Garshasbi and Faghih Zadeh 2005	Weak	Strong	Strong	Weak	Weak	Strong	**Weak**
Ronnberg et al. 2014	Moderate	Strong	Strong	Weak	Strong	Strong	**Moderate**
Stafne et al. 2012	Moderate	Strong	Strong	Weak	Strong	Strong	**Moderate**
Songoygard et al. 2012	Moderate	Strong	Strong	Moderate	Strong	Strong	**Strong**
Gustafsson et al. 2016	Moderate	Strong	Strong	Weak	Weak	Strong	**Weak**
Eggen et al. 2012	Weak	Strong	Weak	Moderate	Strong	Moderate	**Weak**
Miquelutti et al. 2013	Moderate	Strong	Strong	Weak	Strong	Moderate	**Moderate**
Sagedal et al. 2017	Moderate	Strong	Strong	Moderate	Strong	Strong	**Strong**
Haakstad et al. 2018	Moderate	Strong	Strong	Moderate	Strong	Moderate	**Moderate**
Montoya Arizabaleta et al. 2010	Moderate	Strong	Strong	Moderate	Strong	Moderate	**Moderate**
Robledo-Colonia et al. 2012	Moderate	Strong	Strong	Moderate	Strong	Strong	**Strong**
Marquez-Sterling et al. 2000	Weak	Weak	Strong	Weak	Strong	Moderate	**Weak**
Suputtitada et al. 2002	Weak	Weak	Strong	Weak	Strong	Moderate	**Weak**
Hui et al. 2006	Weak	Weak	Strong	Weak	Weak	Moderate	**Weak**
Hui et al. 2012	Strong	Strong	Strong	Weak	Strong	Moderate	**Moderate**
Haakstad and Bo 2011	Weak	Strong	Strong	Moderate	Strong	Moderate	**Moderate**
Haakstad and Bo 2015	Weak	Strong	Strong	Moderate	Weak	Moderate	**Weak**
Haakstad et al. 2016	Weak	Strong	Strong	Moderate	Weak	Weak	**Weak**
Perales et al. 2015	Moderate	Strong	Strong	Moderate	Strong	Strong	**Strong**
da Silva et al. 2017	Moderate	Strong	Strong	Weak	Strong	Strong	**Moderate**
Kluge et al. 2011	Weak	Strong	Weak	Weak	Strong	Strong	**Weak**
Gau et al. 2011	Moderate	Strong	Strong	Weak	Strong	Weak	**Weak**
Aşcı and Rathfisch 2016	Moderate	Strong	Strong	Strong	Strong	Strong	**Strong**
Ruiz et al. 2013	Moderate	Strong	Strong	Weak	Strong	Strong	**Moderate**
Sklempe Kokic et al. 2017	Weak	Strong	Strong	Moderate	Strong	Strong	**Moderate**
Ghodsi and Asltoghiri 2014	Weak	Weak	Strong	Weak	Weak	Weak	**Weak**

EPHPP: the Effective Public Health Practice Project.

**Table 3:** Characteristics of included studies investigating the effect of physical activity interventions on pregnancy-related pain.

Author/Year/Country	Study Design/Settings	Participant Characteristics/Sample Size/Number of Withdrawals	Intervention Components	Interveners	Assessed Outcomes on Pain/Data Collection Time Points	Assessment tools for Outcome Assessment	Findings
Ozdemir et al. 2015; Turkey	Randomized controlled trial; Local hospital in Ankara	Adult pregnant women at 20–35 weeks of gestation *n* = 96 (Intervention: 48, control: 48) ***Withdrawals*** Intervention: 0, control: 0	***Intervention group:*** ***Counselling*** Attendance to 45-min counselling sessions involving education on pregnancy-related low back and pelvic pain, its preventive measures and exercises that can be done during pregnancy, with an educational booklet on these topics provided. ***Unsupervised exercise program*** Participation in an exercise program, comprising mattress exercises (stretching, tightening and loosening) and/or walking exercise. These exercises were to be done at least three times per week, each lasting 30 min, for a period of four weeks. ***Control group:*** Usual care	Nurse	Intensity of low back and pelvic pain, both at rest and during activity Data collected at: Baseline Postintervention	Visual analogue scale	***Within-group comparison*** For the control group, there was no significant change for the perceived pain intensity ratings at rest (*p* = 0.204) or during activity (*p* = 0.258), after the participants received the intervention. However, for the intervention group, a significant decrease in the perceived pain intensity rating at rest (*p* = 0.001) and during activity (*p* = 0.001), after the intervention. ***Between-group comparison*** At the end of the intervention, intervention participants had a significantly lower perceived pain intensity than control participants, for both perceived pain at rest (*p* = 0.001) and that during activity (*p* = 0.001).
Garshasbi and Faghih Zadeh 2005; Iran	Randomized controlled trial; a local hospital in Tehran	Adult first-time pregnant women at 17–22 weeks of gestation *n* = 266 (Intervention: 161, control: 105) ***Withdrawals*** Intervention: 0, control: 0	***Intervention group:*** ***Supervised exercise program*** Attendance to exercise classes involving slow walking, extension movements, anaerobic exercises and specific exercise. These sessions were held three times a week, over a period of 12 weeks. ***Control group:*** Did not receive the intervention	Midwife	Low back pain intensity Data collected at: Baseline Postintervention	The KEBK Questionnaire	***Within-group comparison*** Low back pain among intervention participants was significantly decreased after the intervention (*p* < 0.001) However, low back pain among control participants was significantly increased after the intervention (*p* < 0.001). ***Between-group comparison*** After the intervention, the reported low back pain intensity among the intervention participants was significantly lower than that among control participants. (*p* = 0.006)
Stafne et al. 2012; Norway	Randomized controlled trial; Local hospitals in Trondheim and Stavanger	Adult women with singleton pregnancy, at 18th–22nd week of pregnancy *n* = 855 (Intervention: 429, control: 426) ***Withdrawals*** Intervention: 33, control: 61	***Intervention group:*** ***Supervised exercise program*** Supervised group exercise sessions, each lasting 60 min, held once per week over a 12-week period. Sessions consisted of 30–35 min low-impact aerobics, 20–25 min strength exercises, and 5–10 min of stretching, body awareness, breathing and relaxation exercises. ***Home-based exercise program*** Included a 45-min home exercises twice per week, consisting of endurance training and strength and balance exercises. ***Control group:*** Standard antenatal care	Physiothera-pists	Prevalence of lumbopelvic pain Prevalence of sick leave due to lumbopelvic pain Pain intensity reported in mornings and evenings Data collected at: Baseline (18–22 weeks of pregnancy 32–36 weeks of pregnancy	Visual analog scale Author-developed questionnaire	***Between-group comparison*** At the end of the intervention, there were no difference in the proportion of participants suffering from lumbopelvic pain between the two groups. (adjusted OR: 1.0, 95% CI: 0.7–1.5, *p* = 0.86) However, a significantly smaller proportion of intervention participants had to take sick leave due to lumbopelvic pain at the end of the intervention, compared to control participants (adjusted OR: 0.7, 95% CI: 0.5–1.0, *p* = 0.04) No significant difference was observed in the mean rating of pain intensity experienced in the mornings (*p* = 0.41) or in the evenings (*p* = 0.62) by the participants in the two groups.
Eggen et al. 2012; Norway	Randomized controlled trial; Two local maternity primary care centers in Southeast Norway	Adult pregnant women, before the 20th week of gestation *n* = 257 (Intervention: 129, control: 128) ***Withdrawals*** Intervention: 26, control: 21	***Intervention group:*** ***Supervised exercise program*** Participation in 60-min group exercise sessions once per week, for a period of 16–20 weeks. Exercises focused on the muscles in the lumbopelvic region. ***Information dissemination*** Information provided on normal changes related to pregnancy, and ergonomic advices. ***Home-based exercise program*** Participants were also advised to do suitable exercises at home, highlighting the importance of doing physical exercises with optimal rest in between. These exercises focus on the activation of muscles at the pelvic floor, abdomen, thigh and hips. ***Control group:*** Standard care	Physiothera-pists	Prevalence of low back pain and pelvic girdle pain Pain intensity reported in mornings and evenings Data collected at: Baseline (before 20th week of gestation) 24th week of gestation 28th week of gestation 32th week of gestation 36th week of gestation	Numerical pain rating scale Author-developed questionnaire	***Between-group comparison*** *Prevalence of low back pain and pelvic girdle pain* The intervention had no effect on the prevalence of low back pain (OR: 1.03; 95% CI: 0.66–1.59) or pelvic girdle pain (OR: 0.77; 95% CI: 0.50–1.19) *Pain intensity* Mean difference in pain intensity between intervention and control group participants in the mornings was −0.4 (95% CI: −0.8–0.1). Mean difference in pain intensity between intervention and control group participants in the evenings was −0.4 (95% CI: −1.0–0.2). Therefore, the intervention had no effect on the reduction of pain intensity among the participants.
Miquelutti et al. 2013; Brazil	Randomized controlled trial; A local hospital and four primary healthcare centers in Sao Paulo	Adult women with singleton pregnancy, at 18–24 weeks of gestation *n* = 205 (Intervention: 103, control: 102) ***Withdrawals*** Intervention: 3, control: 1	***Intervention group:*** ***Supervised exercise program*** Attendance to 50-min sessions of nonaerobic exercises that involve the contraction of pelvic floor muscles. ***Counselling *** Oral guidance on pain prevention and control, and information provided to increase participants’ awareness on pelvic floor muscles ***Home-based exercise program*** Participants were also provided with a guide for performing exercises at home. These exercises include pelvic floor muscle training, stretching, exercises to improve venous return in lower limbs, exercises on abdominal muscles and training on progressive relaxation techniques. ***Control group:*** Attendance to educational sessions on breastfeeding, signs and symptoms of labor, as well as visits to delivery ward	Physiothera-pists	Lumbopelvic pain levels Data collected at: 18–24 weeks of pregnancy 28–30 weeks of pregnancy 36–38 weeks of pregnancy	Visual analogue scale	***Between-group comparison*** *Lumbopelvic pain* No significant difference in the prevalence and perceived intensity of lumbopelvic pain between groups at all data collection timepoints. (*p* values not reported)
Suputtitada et al. 2002; Thailand	Randomized controlled trial; Prenatal clinic of a local hospital	Adult first-time pregnant women, at the 26th–30th week of gestation *n* = 84 (Intervention: 42, control: 42) ***Withdrawals*** Total: 7. Number of withdrawals in each group were not specified.	***Intervention group:*** ***Supervised exercise program*** Participation in the ‘sitting pelvic tilt exercise’ program, delivered twice per week at the hospital and three times per week at home. The sessions were held twice per day over a period of eight weeks. ***Control group:*** Did not participate in the exercise program	Exercise instructors	Intensity of back pain Data collected at: Baseline Postintervention	Visual analog scale	***Between-group comparison*** Most (90.6%) of the intervention participants expressed that the intensity of back pain was improved by the end of the intervention, while most (94.3%) of the control participants reported worsened pain intensity. At the end of the intervention, the mean rating of back pain intensity among the intervention participants was significantly lower than that among control participants (*p* < 0.001)
Haakstad and Bo 2015; Norway	Secondary analysis of randomized controlled trial; Local community	First-time pregnant women before the 24th week of pregnancy *n* = 105 (Intervention: 52, control: 53) ***Withdrawals*** Intervention: 10, control: 11	***Intervention group:*** ***Supervised exercise program*** Supervised 1-hour sessions of moderate-intensity aerobic dance at least twice per week for 12 weeks. The exercises emphasized cardiovascular endurance training and strength training. Aerobic dance sessions comprise 5-min warm up exercise, 35 min of endurance training and aerobic dance, 15 min of strength training of deep abdominal stabilization muscles, pelvic floor and back muscles, followed by 5 min of stretching, relaxation and body awareness exercises. Music was played during these sessions. ***Unsupervised exercise program*** 30-minsessions of self-imposed moderate-intensity physical activity. ***Control group:*** Asked to practice their usual physical activity habits	Aerobic instructors	Number of participants reporting pelvic girdle pain and low back pain Data collected at: Baseline Postintervention Postpartum period (6–8 weeks after delivery)	Author-developed questionnaire	***Between-group comparison*** *Intention-to-treat analysis* After the intervention, no difference was observed in the number of participants reporting pelvic girdle pain (OR: 0.81; 95% CI: 0.37–1.78; *p* = 0.60) or low back pain (OR: 2.60; 95% CI: 0.27–25.99; *p* = 0.95) *Per-protocol analysis* After the intervention, no difference was observed in the number of participants reporting pelvic girdle pain (OR: 1.21; 95% CI: 0.76–1.92; *p* = 0.43) or low back pain (OR: 1.00; 95% CI: 0.16–6.26; *p* = 0.90) *Subgroup analysis with participants with 100% compliance with the exercise program* After the intervention, no difference was observed in the number of participants reporting pelvic girdle pain (OR: 1.16; 95% CI: 0.75–1.79; *p* = 0.51) or low back pain (OR: 1.74; 95% CI: 0.33–9.19; *p* = 0.51)
Kluge et al. 2011; South Africa	Randomized controlled trial; prenatal clinics at two local hospitals in Western Cape	Adult pregnant women with a gestational age of 16–24 weeks, who were experiencing low back pain *n* = 50 (Intervention: 26, control: 24) ***Withdrawals*** Intervention: 2, control: 2	***Intervention group:*** ***Information dissemination*** Provided with verbal and written information (in a pamphlet) on basic back care. ***Supervised exercise program*** Attendance to exercise classes that were held once every two weeks for ten weeks, each class lasted for 30–45 min. Exercise classes involved stretch exercises, relaxation and breathing techniques, with the exercises focusing on the abdominal muscles and pelvic floor muscles ***Telephone reminders*** Telephone reminders to participants, encouraging them to do regular exercise at home. ***Control group:*** Did not receive the intervention	Biokineticist and the investigator of the study	Pain intensity Data collected at: Baseline Postintervention	Brief Pain Inventory	***Within-group comparison*** A significant reduction in perceived pain intensity among the intervention participants at postintervention (*p* < 0.01). A slight yet nonsignificant perceived pain intensity among the control participants at postintervention (*p* = 0.89) ***Between-group comparison*** The perceived pain intensity among the intervention participants was significantly lower than that among the control participants at postintervention (*p* < 0.01).
Gau et al. 2011; Taiwan	Randomized controlled trial; Local hospital and medical center in Taiwan	Adult women with singleton pregnancy, at 30–32 weeks of gestation *n* = 188 (Intervention: 94, control: 94) ***Withdrawals*** Intervention: 46, control: 55	***Intervention group:*** ***Home-based exercise program*** The birth ball exercise program, involving the use of birth ball for exercises in positions including sitting, standing, kneeling and squatting. Exercises to be carried out at home three times per week, in sessions of at least 20 min, for a period of 6–8 weeks. A booklet and video tape were provided showing the procedures of the exercises involved in the program. ***Control group:*** Standard nursing and midwifery care	Investigators of the study	Labor pain Data collected when: Cervical dilation of participants reached 4 cm Cervical dilation of participants reached 8 cm	Short form McGill Pain Questionnaire (SF-MPQ)	***Between-group comparisons*** At both the time points when cervical dilation of the participants reached 4 cm and 8 cm, participants in the intervention group reported a significantly lower pain score than those in the control group (*p* ≤ 0.002)
Sklempe Kokic et al. 2017; Croatia	Secondary analysis of randomized controlled trial; Two local hospitals in Zagreb	Adult pregnant women before 30 weeks of gestation *n* = 45 (Intervention: 22, control: 23) ***Withdrawals*** Intervention: 2, control: 1	***Intervention group:*** ***Supervised exercise program*** Participation in an individual supervised exercise program, with sessions held twice per week, each lasting 50–55 min, for a duration of six week or more (throughout the participants’ pregnancy). The exercise sessions consisted of aerobic exercises on a treadmill, resistance exercises, pelvic floor exercises, stretching and relaxation. ***Unsupervised exercise program*** Participants were also asked to do brisk walking for 30 min each day ***Control group:*** Standard antenatal care	Not specified	Number of participants reporting lumbopelvic pain Lumbopelvic pain intensity Data collected at: Baseline Postintervention	Numeric rating scale (NRS) Pelvic Girdle Questionnaire (PGQ)	***Between-group comparison*** *Lumbopelvic pain* A lower proportion of intervention participants developed lumbopelvic pain after the intervention, compared to control participants, but the difference was not significant (55.0% vs. 81.8%; *p* = 0.064). Lower intensity of lumbopelvic pain was experienced by intervention participants after the intervention compared to controls, as evidenced by the significantly lower NRS score (*p* = 0.017) and PGQ score (*p* = 0.017).

**Table 4:** Characteristics of included studies investigating the effect of physical activity interventions on gestational weight gain.

Author/Year/Country	Study Design/Settings	Participant Characteristics/Sample Size/Number of Withdrawals	Intervention Components	Interveners	Assessed Outcomes on Gestational Weight Gain/Data Collection Time Points	Assessment Tools for Outcome Assessment	Findings
Kinnunen et al. 2007; Finland	Controlled clinical trial; Six maternity clinics in southern Finland	Adult, first-time pregnant women *n* = 122 (Intervention: 69, control: 53) ***Withdrawals*** Intervention: 20, control: 7	**Comprising dietary and physical activity components** *Physical activity components* ***Intervention group:*** ***Counselling*** Counselling sessions with the aim to increase the participants’ leisure time physical activity (LTPA) levels, and achieve the recommended level of LTPA levels. ***Supervised exercise program (optional)*** Optional attendance to supervised exercise sessions lasting for 45–60 min, with sessions held once per week. ***Control group:*** Standard maternity care	Public health nurses	Gestational weight gain Data collected at: Baseline Before the first booster session Postintervention	Weight measurement using a scale	***Between-group comparisons*** No significant difference was observed in gestational weight gain among participants in the two groups (*p* = 0.77). The intervention had no effect on preventing excessive gestational weight gain among the participants (adjusted OR = 1.82, 95% CI: 0.65–5.14, *p* = 0.26)
Huang et al. 2011; Taiwan	Three-group randomized controlled trial; clinic at a local medical center in northern Taiwan	Adult women before the 16th week of gestation *n* = 240 (First intervention group (EP): 80, second intervention group (EPP): 80, control group: 80) ***Withdrawals*** EP: 19, EPP: 16control: 16	**Comprising dietary and physical activity components** *Physical activity components* ***First intervention group (EP)*** ***Counselling and information dissemination*** Six sessions of individual counselling, focusing on goal setting in physical activity behaviors. A brochure containing information on the recommended exercise types and their benefits was also provided. The intervention lasted from the 16th gestational week to 6 months postpartum. ***Second intervention group (EPP)*** Same intervention as the EP group, but it lasted from 24–48 h after delivery to 6 months post-partum and consisted of only three counselling sessions. ***Control group*** Usual care	Nurse	Gestational weight gain Data collected at: Baseline Six months postpartum	Not specified	***Between-group comparisons*** Both gestational weight gain and postpartum weight retention among the participants in the two intervention groups was significantly smaller than that in the control group (*p* < 0.001).
Garshasbi and Faghih Zadeh 2005; Iran	Randomized controlled trial; a local hospital in Tehran	Adult first-time pregnant women at 17–22 weeks of gestation *n* = 266 (Intervention: 161, control: 105) ***Withdrawals*** Intervention: 0, control: 0	***Intervention group:*** ***Supervised exercise program*** Attendance to exercise classes involving slow walking, extension movements, anaerobic exercises and specific exercise. These sessions were held three times a week, over a period of 12 weeks. ***Control group:*** Did not receive the intervention.	Midwife	Maternal weight gain	The KEBK Questionnaire	***Between-group comparison*** No significant difference in the level of maternal weight gain during pregnancy between the two groups of participants (*p* = 0.63)
Ronnberg et al. 2014; Sweden	Randomized controlled trial; antenatal clinics in the Orebro County of Sweden	Adult pregnant women on or before their 16th week of pregnancy *n* = 445 (Intervention: 221, control: 224) ***Withdrawals*** Intervention: 29, control: 42	***Intervention group:*** ***Information dissemination*** Sessions involving education and information provision on recommended gestational weight gain during pregnancy. ***Unsupervised exercise program*** A formal prescription of physical activity program for the participants, with a duration of 30 min, which was to be carried out each day. ***Control group:*** Standard maternity care	Midwife	Mean gestational weight gain Data collected at: Baseline Date of delivery of participants	Not specified	***Between-group comparisons*** A significantly lower mean gestational weight gain among participants in the intervention group (14.2 kg ± 4.4), compared to those in control group (15.3 kg ± 5.4), (*p* = 0.029) The program also reduced the proportion of participants with gestational weight gain above the Institute of Medicine (IOM) guidelines (Intervention: 41.1%; control: 50.0%), but difference was not significant (*p* = 0.086)
Sagedal et al. 2017; Norway	Randomized controlled trial; Eight healthcare clinics in southern Norway	Adult women with a singleton pregnancy at no more than 20 weeks of gestation *n* = 606 (Intervention: 303, control: 303) ***Withdrawals*** Intervention: 34, control: 39	**Comprising dietary and physical activity components** *Physical activity components* ***Intervention group:*** ***Supervised exercise program*** Supervised group exercise sessions twice per week, each lasting for 60 min, consisting 10 min warm-up, 40 min moderate-intensity cardiovascular and strength training and 10 min stretching. ***Information dissemination*** Booklets are also provided to provide information on recommendations for healthy lifestyles. Further reinforcement of participants’ knowledge on healthy lifestyles is achieved through access to a website containing health information. ***Control group:*** Standard prenatal care	Physiotherapists and students at fitness centers	Gestational weight gain Data collected at: Baseline 36 weeks after gestation	Self-reported pre-pregnancy weight Weighing on admission to delivery ward	***Between-group comparisons*** *Gestational weight gain from pre-pregnancy to term delivery* The gestational weight gain among intervention participants is significantly smaller than that among control participants (mean difference = 1.3 kg; *p* = 0.009). Subgroup analysis showed that the difference is significant among normal weight participants (mean difference = 1.1 kg; *p* = 0.036), but not among overweight (mean difference = 1.4 kg; *p* = 0.321) or obese (mean difference = 3.1 kg; *p* = 0.221) participants. *Gestational weight gain from subject inclusion to the study to term delivery* The gestational weight gain among intervention participants is significantly smaller than that among control participants (mean difference = 0.9 kg; *p* = 0.043). However, subgroup analysis revealed the difference is insignificant among normal weight, overweight and obese participants (*p* > 0.05)
Marquez-Sterling et al. 2000; USA	Randomized controlled trial; Local community	Adult women during their second trimester of pregnancy *n* = 20 (Intervention: 10, Control: 10) ***Withdrawals*** Intervention: 1, control: 4	***Intervention group:*** ***Supervised exercise program*** Attendance to a training program consisting of 1-hour sessions held three times per week for 15 weeks. Training consists of aerobic exercises including rowing, stationary cycling, walk-jogging, brisk walking, and calisthenic exercises. ***Control group:*** Intervention provided at the participants’ postpartum period	Aerobic instructors	Maternal weight gain Skin-fold thickness Data collected at: Baseline Postintervention	Weight measurement using a scale Skinfold caliper	***Between-group comparisons*** No significant differences in maternal weight gain (*p* = 0.649) or skinfold thickness (*p* = 0.843) between participants in the two groups.
Hui et al. 2006; Canada	Pilot randomized controlled trial; Local community in urban Winnipeg	Pregnant women before the 26th week of pregnancy *n* = 52 (Numbers of participants randomized into the two groups are not reported) ***Withdrawals*** Intervention: Not reported, control: Not reported	**Comprising dietary and physical activity components** *Physical activity components* ***Intervention group:*** ***Supervised exercise program*** Participation in an aerobic exercise program with 3–5 exercise sessions (one at the community centers as a group session and the remaining at home) per week, each lasting 30–45 min for a period of 10–16 weeks. ***Control group:*** Standard prenatal care	Fitness instructors	Weight gain during pregnancy Data collected at: Baseline Postintervention	Not specified	***Between-group comparison*** No between-group difference was observed for the extent of weight gain during pregnancy among the participants (*p* = 1.00)
Hui et al. 2012; Canada	Randomized controlled trial; Local community in Winnipeg	Pregnant women before the 26th week of pregnancy *n* = 224 (Intervention: 112, control: 112) ***Withdrawals*** Intervention: 10, control: 24	**Comprising dietary and physical activity components** *Physical activity components* ***Intervention group:*** ***Supervised exercise program*** Participation in an aerobic exercise program with 3–5 exercise sessions (one at the community centers as a group session and the remaining at home) per week, each lasting 30–45 min for a period of 10–16 weeks. ***Control group:*** Standard prenatal care	Fitness instructors	Prevalence of excessive gestational weight gain Gestational weight gain Data collected at: Baseline Two months after being enrolled	Hospital records	***Between-group comparison*** No significant difference in gestational weight gain between the two groups (*p* = 0.28) However, the proportion of participants with excessive gestational weight gain was significantly lower for the intervention group, compared to the control group (*p* = 0.008)
Haakstad and Bo 2011; Norway	Randomized controlled trial; Local community	Adult women within the first 24 weeks of pregnancy *n* = 105 (Intervention: 52, Control: 53) ***Withdrawals*** Intervention: 10, control: 11	***Intervention group:*** ***Supervised exercise program*** Supervised 1-hour sessions of aerobic dance at least twice per week for 12 weeks. The exercises emphasized cardiovascular endurance training and strength training. Aerobic dance sessions comprise 5-min warm up exercise, 35 min of endurance training and aerobic dance, 15 min of strength training of deep abdominal stabilization muscles, pelvic floor and back muscles, followed by 5 min of stretching, relaxation and body awareness exercises. Music was played during these sessions. ***Unsupervised exercise program*** 30-min sessions of self-imposed moderate-intensity physical activity. ***Control group:*** Asked to practice their usual physical activity habits	Aerobic instructors	Maternal weight gain Skin-fold thickness Data collected at: Baseline Postintervention Post-partum (6–12 weeks after delivery)	Weight measurement using a scale Skinfold caliper	***Between-group comparisons*** *Intent-to-treat analysis* No significant difference in maternal weight gain (*p* = 0.31), changes in skinfold thickness (*p* = 0.38) nor mean weight retention (*p* = 0.93) between the two groups. *Analysis by including participants attending more than 80% of the exercise sessions only (per-protocol analysis)* No significant difference in maternal weight gain (*p* = 0.23), nor changes in skinfold thickness (*p* value not reported) between the two groups. Results on postpartum weight are not presented. *Analysis by including participants attending all of the exercise sessions only* Maternal weight gain among participants in the intervention group is significantly lower than those in control group (*p* = 0.01) No significant difference was observed in skinfold thickness among participants in the two groups (*p* value not reported) Significantly lower postpartum weight was observed among participants in the intervention group (*p* = 0.001)
Perales et al. 2015; Spain	Randomized controlled trial; University Hospital of Fuenlabrada in Madrid	Adult women with uncomplicated and singleton gestations *n* = 184 (Intervention: 101, control: 83) ***Withdrawals*** Intervention: 11, control: 6	***Intervention group:*** ***Supervised exercise program*** Supervised physical conditioning program of light- to moderate-intensity aerobic activity three times per week, each lasting 55–60 min. It comprises 5–8 min of walking and muscle stretching, followed by aerobic dance, exercises targeting muscles on the legs, buttocks and abdomen, pelvic floor muscle training and balancing exercises, and a cool-down session. ***Control group:*** Not specified	Qualified fitness specialists	Maternal weight gain Data collected at: Week 9–12 of pregnancy (First trimester) Week 38–39 of pregnancy (Third trimester)	Medical records at delivery	***Between-group comparisons*** Percentage of participants with excessive weight gain during pregnancy is significantly higher in control group compared to intervention group (*p* = 0.034).
da Silva et al. 2017; Brazil	Randomized controlled trial; Health facilities offering antenatal care in Pelotas, Brazil	Adult pregnant women living in urban areas *n* = 639 (Intervention: 213, control: 426) ***Withdrawals*** Intervention: 15, control: 30	***Intervention group:*** ***Supervised exercise program*** A moderate-intensity exercise program that was individually supervised, held 3 days a week for at least 16 weeks. Each session lasting an hour. The program comprises a warm-up session, sessions of aerobic activities, strength training/floor exercises and stretching exercises. ***Control group:*** Standard antenatal care	Trained physical education professionals	Gestational weight gain Data collected at: Baseline (before 20 weeks of gestation) First follow-up (before 28 weeks of gestation) Second follow-up (before 36 weeks of gestation)	Weight measurement	***Between-group comparisons*** Although the intervention group exhibited a smaller extent of gestational weight gain compared to the control group after the intervention, the between-group difference was found not to be statistically significant using intention-to-treat analysis (*p* = 0.10), per protocol analysis with 70% adherence as limitation (*p* = 0.10) or per protocol analysis with 100% adherence as limitation (*p* = 0.31)
Aşcı and Rathfisch 2016; Turkey	Randomized controlled trial; Local family health center in Istanbul	Adult pregnant women who were pregnant for less than three months *n* = 102 (Intervention: 51, control: 51) ***Withdrawals*** Intervention: 6, control: 6	**Comprising dietary and physical activity components** *Physical activity components* ***Intervention group:*** ***Counselling*** Interviews, each lasting 1 h, for educating participants on the importance on healthy lifestyle, and making recommendations on the low-level aerobic exercises, encouraging them to do moderate-intensity physical exercises regularly. ***Control group:*** Standard care	Investigator of the study	Gestational weight gain Data collected at: Baseline Postintervention 6 weeks at postpartum	Weight measurement Health Promoting Lifestyle Profile II (HPLP-II)	***Between-group comparison*** *Gestational weight gain* No significant difference in gestational weight gain between groups at postintervention (*p* = 0.87) Nevertheless, proportion of participants in the intervention group with gestational weight gain within the limit recommended by IOM is significantly higher than that in control group. (*p* = 0.03)
Ruiz et al. 2013; Spain	Randomized controlled trial; Local primary care medical centers in Madrid	Women with singleton pregnancy, at the 5th–6th week of gestation *n* = 962 (Intervention: 481, control: 481) ***Withdrawals*** Intervention: 70, control: 68	***Intervention group:*** ***Supervised exercise program*** Participation in a light- to moderate-intensity exercise program, involving group exercise sessions lasting 50–55 min, three times per week for 30 weeks. Sessions involved both aerobic, flexibility and resistance exercises. ***Control group:*** Standard care	Not specified	Gestational weight gain Data collected at: Baseline Postintervention	Weight measurement	***Between-group comparison*** Overall, intervention participants exhibited significantly lower gestational weight gain than control participants (*p* < 0.001) Among the normal weight participants, the difference in gestational weight gain between intervention group and control group was significant (*p* < 0.001), but the difference was not significant among overweight or obese participants (*p* = 0.51) Overall, intervention participants were significantly less likely to gain weight that is above the Institute of Medicine recommendations, compared to controls (*p* = 0.002) This significant difference was observed among participants with normal weight (*p* = 0.002), but not among overweight or obese participants (*p* = 0.14)
Ghodsi and Asltoghiri 2014; Iran	Randomized controlled trial; Prenatal clinics and delivery centers in Hamedan, Iran	Adult pregnant women at 20–26 weeks of gestation *n* = 80 (Intervention: 40, control: 40) ***Withdrawals*** Intervention: Not reported control: Not reported	***Intervention group:*** ***Home-based exercise program*** A program of cycling at participants’ home on a bicycle ergometer three times per week, with each session lasting 15 min. Participants were encouraged to do the cycling at home. ***Control group:*** Did not receive the intervention	Not specified	Gestational weight gain Data collected at: Baseline Postintervention	Records at prenatal clinics	***Between-group comparison*** There was no significant difference in the gestational weight gain between the intervention and control groups (*p* = 0.14)

**Table 5:** Characteristics of included studies investigating the effect of physical activity interventions on psychological outcomes.

Author/Year/Country	Study Design/Settings	Participant Characteristics/Sample Size/Number of Withdrawals	Intervention Components	Interveners	Assessed Outcomes on Gestational Weight Gain/Data Collection Time Points	Assessment Tools for Outcome Assessment	Findings
Huang et al. 2011; Taiwan	Three-group randomized controlled trial; clinic at a local medical center in northern Taiwan	Adult women before the 16th week of gestation *n* = 240 (First intervention group (EP): 80, second intervention group (EPP): 80, control group: 80) ***Withdrawals*** EP: 19, EPP: 16 control: 16	**Comprising dietary and physical activity components** *Physical activity components* ***First intervention group (EP)*** ***Counselling and information dissemination*** Six sessions of individual counselling, focusing on goal setting in physical activity behaviors. A brochure containing information on the recommended exercise types and their benefits was also provided. The intervention lasted from the 16th gestational week to 6 months postpartum. ***Second intervention group (EPP)*** Same intervention as the EP group, but it lasted from 24–48 h after delivery to 6 months post-partum and consisted of only three counselling sessions. ***Control group*** Usual care	Nurse	Depression Data collected at: Baseline Six months postpartum	Beck Depression Inventory	***Between-group comparisons*** Participants in the first intervention group (EP group) exhibited a significantly lower extent of increase in the depression score compared to those in the second intervention group (EPP group) and control group (*p* < 0.001)
Songoygard et al. 2012; Norway	Randomized controlled trial; Local hospitals in Trondheim and Stavanger	Adult pregnant women attending ultrasound examination during the 18th week of pregnancy *n* = 855 (Intervention: 429, control: 426) ***Withdrawals*** Intervention: 50, control: 86	***Intervention group:*** ***Supervised exercise program*** Supervised group exercise sessions, each lasting 60 min, held once per week over a 12-week period. Sessions consisted of 30–35 min low-impact aerobics, 20–25 min strength exercises, and 5–10 min of stretching, body awareness, breathing and relaxation exercises. ***Home-based exercise program*** Included a 45-min home exercises twice per week, consisting of endurance training and strength and balance exercises. ***Control group:*** Standard antenatal care	Physiotherapists	Depression Data collected at: Baseline Postintervention 3-month postpartum	Edinburgh Postnatal Depression Scale (EPDS)	***Between-group comparisons*** At 3-month postpartum, no difference in the EPDS score was observed between the intervention and control groups (intervention: 2.52 ± 2.90; control: 2.52 ± 3.30); *p* = 0.35. Among participants who exhibited compliance to the intervention only, no difference in EPDS score was observed between the two groups (*p* = 0.79). ***Subgroup analysis*** By including only participants who did not do exercise before pregnancy in the analysis, the intervention was found to cause a significant reduction in the proportion of participants with an EPDS score of 10 or more (*p* = 0.03). However, the intervention did not cause a significant reduction in the proportion of participants with an EPDS score of 13 or more (*p* = 0.11)
Gustafsson et al. 2016; Norway	Randomized controlled trial; Local hospitals in Trondheim and Stavanger	Adult pregnant women attending ultrasound examination during the 18th week of pregnancy *n* = 855 (Intervention: 429, control: 426) ***Withdrawals*** Intervention: 33, control: 61	***Intervention group:*** ***Supervised exercise program*** Supervised group exercise sessions, each lasting 60 min, held once per week over a 12-week period. Sessions consisted of 30–35 min low-impact aerobics, 20–25 min strength exercises, and 5–10 min of stretching, body awareness, breathing and relaxation exercises. ***Home-based exercise program*** Included a 45-min home exercises twice per week, consisting of endurance training and strength and balance exercises. ***Control group:*** Standard antenatal care	Physiotherapists	Psychological well-being (anxiety and depression) Data collected at: Baseline Postintervention	Psychological General Well-being Index	***Between-group comparisons*** After the intervention, no significant differences were observed between groups in all of the outcomes investigated, including anxiety (*p* = 0.23) and depressed mood (*p* = 0.90).
Miquelutti et al. 2013; Brazil	Randomized controlled trial; A local hospital and four primary healthcare centers in Sao Paulo	Adult women with singleton pregnancy, at 18–24 weeks of gestation *n* = 205 (Intervention: 103, control: 102) ***Withdrawals*** Intervention: 3, control: 1	***Intervention group:*** ***Supervised exercise program*** Attendance to 50-min sessions of nonaerobic exercises that involve the contraction of pelvic floor muscles. ***Counselling *** Oral guidance on pain prevention and control, and information provided to increase participants’ awareness on pelvic floor muscles ***Home-based exercise program*** Participants were also provided with a guide for performing exercises at home. These exercises include pelvic floor muscle training, stretching, exercises to improve venous return in lower limbs, exercises on abdominal muscles and training on progressive relaxation techniques. ***Control group:*** Attendance to educational sessions on breastfeeding, signs and symptoms of labor, as well as visits to delivery ward	Physiotherapists	Anxiety Data collected at: 18–24 weeks of pregnancy 28–30 weeks of pregnancy 36–38 weeks of pregnancy	State-trait anxiety inventory	***Between-group comparison*** No significant difference in anxiety levels was observed between groups at all data collection timepoints. (*p* values not reported)
Robledo-Colonia et al. 2012; Columbia	Randomized controlled trial; Three local hospitals in Cali	Adult pregnant women at 16–20 weeks of gestation *n* = 80 (Intervention: 40, control: 40) ***Withdrawals*** Intervention: 3, control: 3	***Intervention group:*** ***Supervised exercise program*** Group exercise classes held three times per week, each lasting for 60 min, for a period of three months. These sessions consist of walking, aerobic exercises, stretching and relaxation ***Control group:*** Did not attend the exercise classes	Physiotherapists and physicians	Depression Data collected at: Baseline Postintervention (3 months after baseline data collection)	Center for Epidemiological Studies-Depression Scale (CES-D)	***Between-group comparisons*** The intervention induced a more significant decrease in the CES-D score among the intervention participants, compared to controls. A difference of four points in the reduction in CES-D score was observed between intervention and control groups.
Haakstad et al. 2016; Norway	Secondary analysis of randomized controlled trial; Local community	Adult women within the first 24 weeks of pregnancy *n* = 105 (Intervention: 52, Control: 53) ***Withdrawals*** Intervention: 0, control: 0	***Intervention group:*** ***Supervised exercise program*** Supervised 1-hour sessions of aerobic dance at least twice per week for 12 weeks. The exercises emphasized cardiovascular endurance training and strength training. Aerobic dance sessions comprise 5-min warm up exercise, 35 min of endurance training and aerobic dance, 15 min of strength training of deep abdominal stabilization muscles, pelvic floor and back muscles, followed by 5 min of stretching, relaxation and body awareness exercises. Music was played during these sessions. ***Unsupervised exercise program*** 30-min sessions of self-imposed moderate-intensity physical activity. ***Control group:*** Asked to practice their usual physical activity habits	Aerobics instructors	Maternal pregnancy depression Data collected at: Baseline Postintervention	WHOQOL-BREF SF-36	***Between-group comparisons*** *Intention-to-treat analysis* No significant difference in the score for the frequency of having negative mood feelings such as sadness, despair, anxiety and depression between groups at postintervention, where higher score indicates lower frequency of such feelings (*p* = 0.4) No significant difference in the proportion of participants reporting pregnancy depression between groups at postintervention. (*p* = 0.07) *Per-protocol analysis with participants exhibiting 100% exercise adherence* Significant difference was observed in the score for the frequency of having negative mood feelings between groups at postintervention (*p* = 0.01) Difference in the proportion of participants reporting pregnancy depression between groups did not reach statistical significance at postintervention (*p* = 0.4)
Perales et al. 2015; Spain	Randomized controlled trial; University Hospital of Fuenlabrada in Madrid	Adult women with uncomplicated and singleton gestations *n* = 184 (Intervention: 101, control: 83) ***Withdrawals*** Intervention: 11, control: 6	***Intervention group:*** ***Supervised exercise program*** Supervised physical conditioning program of light- to moderate-intensity aerobic activity three times per week, each lasting 55–60 min. It comprises 5–8 min of walking and muscle stretching, followed by aerobic dance, exercises targeting muscles on the legs, buttocks and abdomen, pelvic floor muscle training and balancing exercises, and a cool-down session. ***Control group:*** Not specified	Qualified fitness specialists	Depression during pregnancy Data collected at: Week 9–12 of pregnancy (First trimester) Week 38–39 of pregnancy (Third trimester)	Center for Epidemiological Studies—Depression scale (CES-D)	***Within-group comparisons*** For intervention group, a significant decrease in depression score was observed at postintervention. (*p* = 0.001). A small effect size was observed (Cohen’s *d* = 0.29) For control group, a significant increase in depression score was observed at postintervention. (*p* = 0.039). A small effect size was observed (Cohen’s *d* = 0.22) ***Between-group comparisons*** Significantly lower depression score was observed among the intervention participants compared to the control participants at postintervention (*p* = 0.005), while no significant difference in depression score was observed between groups at baseline (*p* = 0.71). A small effect size was observed (Cohen’s *d* = 0.46)

**Table 6:** Characteristics of included studies investigating the effect of physical activity interventions on quality of life.

Author/Year/Country	Study Design/Settings	Participant Characteristics/Sample Size/Number of Withdrawals	Intervention Components	Interveners	Assessed Outcomes on Gestational Weight Gain/Data Collection Time Points	Assessment Tools for Outcome Assessment	Findings
Montoya Arizabaleta et al. 2010; Columbia	Randomized controlled trial; Three local hospitals in Cali	Adult pregnant women at 16–20 weeks of gestation *n* = 64 (Intervention: 33, control: 31) ***Withdrawals*** Intervention: 9, control: 5	***Intervention group:*** ***Supervised exercise program*** Group exercise classes held three times per week, each lasting for 60 min, for a period of three months. These sessions consist of walking, aerobic exercises, stretching and relaxation ***Control group:*** Did not attend the exercise classes	Physiotherapists and physicians	Health-related quality of life Data collected at: Baseline Postintervention (3 months after baseline data collection	Colombian version of the Medical Outcome Study Short-Form Health Survey (SF-12)	***Between-group comparisons*** After the intervention, the score of 3 of the 4 domains in the physical component of SF-12 was increased significantly more among the intervention participants, compared to controls. These include the physical function domain (7-point difference), bodily pain domain (7-point difference) and general health domain (5-point difference). No significant difference in the increase of the score in the role physical domain (1-point difference) The intervention caused no significant difference in the increase in the score of the domains in the mental component of SF-12 between the two groups.
Haakstad et al. 2016; Norway	Secondary analysis of randomized controlled trial; Local community	Adult women within the first 24 weeks of pregnancy *n* = 105 (Intervention: 52, Control: 53) ***Withdrawals*** Intervention: 0, control: 0	***Intervention group:*** ***Supervised exercise program*** Supervised 1-h sessions of aerobic dance at least twice per week for 12 weeks. The exercises emphasized cardiovascular endurance training and strength training. Aerobic dance sessions comprise 5-min warm up exercise, 35 min of endurance training and aerobic dance, 15 min of strength training of deep abdominal stabilization muscles, pelvic floor and back muscles, followed by 5 min of stretching, relaxation and body awareness exercises. Music was played during these sessions. ***Unsupervised exercise program*** 30-min sessions of self-imposed moderate-intensity physical activity. ***Control group:*** Asked to practice their usual physical activity habits	Aerobics instructors	Maternal pregnancy depression Data collected at: Baseline Postintervention	World Health Organization Quality of Life Instrument, Short Form (WHOQOL-BREF) The Short Form (36) Health Survey (SF-36)	***Between-group comparisons*** *Intention-to-treat analysis* No significant difference in the quality of life sum score between groups at postintervention (*p* = 0.3) *Per-protocol analysis with participants exhibiting more than 80% exercise adherence* No significant difference in the quality of life sum score between groups at postintervention (*p* = 0.8) *Per-protocol analysis with participants exhibiting more than 100% exercise adherence* No significant difference in the quality of life sum score between groups at postintervention (*p* = 0.2)

**Table 7:** Characteristics of included studies investigating the effect of physical activity interventions on physical activity levels and self-efficacy.

Author/Year/Country	Study Design/Settings	Participant Characteristics/Sample Size/Number of Withdrawals	Intervention Components	Interveners	Assessed Outcomes on Gestational Weight Gain/Data Collection Time Points	Assessment Tools for Outcome Assessment	Findings
Kinnunen et al. 2007; Finland	Controlled clinical trial; Six maternity clinics in southern Finland	Adult, first-time pregnant women *n* = 122 (Intervention: 69, control: 53) ***Withdrawals*** Intervention: 20, control: 7	**Comprising dietary and physical activity components** *Physical activity components* ***Intervention group:*** ***Counselling*** Counselling sessions with the aim to increase the participants’ leisure time physical activity (LTPA) levels, and achieve the recommended level of LTPA. ***Supervised exercise program (optional)*** Optional attendance to supervised exercise sessions lasting for 45–60 min, with sessions held once per week. ***Control group:*** Standard maternity care	Public health nurses	Leisure time physical activity levels Data collected at: Baseline Before the first booster session Postintervention	International Physical Activity Questionnaire (IPAQ)	***Between-group comparisons*** No significant difference was observed in the leisure time physical activity level (*p* value not reported).
Huang et al. 2011; Taiwan	Three-group randomized controlled trial; clinic at a local medical center in northern Taiwan	Adult women before the 16th week of gestation *n* = 240 (First intervention group (EP): 80, second intervention group (EPP): 80, control group: 80) ***Withdrawals*** EP: 19, EPP: 16 control: 16	**Comprising dietary and physical activity components** *Physical activity components* ***First intervention group (EP)*** ***Counselling and information dissemination*** Six sessions of individual counselling, focusing on goal setting in physical activity behaviors. A brochure containing information on the recommended exercise types and their benefits was also provided. The intervention lasted from the 16th gestational week to 6 months postpartum. ***Second intervention group (EPP)*** Same intervention as the EP group, but it lasted from 24–48 h after delivery to 6 months post-partum and consisted of only three counselling sessions. ***Control group*** Usual care	Nurse	Self-efficacy in physical activity Data collected at: Baseline Six months postpartum	Self-rated Abilities for Health Practices Scale	***Between-group comparisons*** Increase in self-efficacy in physical activity among participants in the two intervention groups was significantly greater, compared to those in control group (*p* < 0.001).
Miquelutti et al. 2013; Brazil	Randomized controlled trial; A local hospital and four primary healthcare centers in Sao Paulo	Adult women with singleton pregnancy, at 18–24 weeks of gestation *n* = 205 (Intervention: 103, control: 102) ***Withdrawals*** Intervention: 3, control: 1	***Intervention group:*** ***Supervised exercise program*** Attendance to 50-min sessions of nonaerobic exercises that involve the contraction of pelvic floor muscles. ***Counselling *** Oral guidance on pain prevention and control, and information provided to increase participants’ awareness on pelvic floor muscles. ***Home-based exercise program*** Participants were also provided with a guide for performing exercises at home. These exercises include pelvic floor muscle training, stretching, exercises to improve venous return in lower limbs, exercises on abdominal muscles and training on progressive relaxation techniques. ***Control group:*** Attendance to educational sessions on breastfeeding, signs and symptoms of labor, as well as visits to delivery ward	Physiotherapists	Physical activity levels Data collected at: 18–24 weeks of pregnancy 28–30 weeks of pregnancy 36–38 weeks of pregnancy	Pregnancy Physical Activity Questionnaire	***Between-group comparison*** Significant difference was observed in physical activity levels between the two groups (*p* = 0.009), with physical activity levels of intervention participants increasing, and that of control participants decreasing, during the study.
Sagedal et al. 2017; Norway	Randomized controlled trial; Eight healthcare clinics in southern Norway	Adult women with a singleton pregnancy at no more than 20 weeks of gestation *n* = 606 (Intervention: 303, control: 303) ***Withdrawals*** Intervention: 34, control: 39	**Comprising dietary and physical activity components** *Physical activity components* ***Intervention group:*** ***Supervised exercise program*** Supervised group exercise sessions twice per week, each lasting for 60 min, consisting 10 min warm-up, 40 min moderate-intensity cardiovascular and strength training and 10 min stretching. ***Information dissemination*** Booklets are also provided to provide information on recommendations for healthy lifestyles. Further reinforcement of participants’ knowledge on healthy lifestyles is achieved through access to a website containing health information. ***Control group:*** Standard prenatal care	Physiotherapists and students at fitness centers	Physical activity level Data collected at: Baseline 36 weeks after gestation	International Physical Activity Questionnaire—short form	***Between-group comparisons*** No significant difference in weekly energy expenditure between groups at baseline (*p* = 0.828), but intervention participants reported significantly higher weekly energy expenditure than control participants at 36 weeks of gestation (*p* = 0.009).
Haakstad et al. 2018; Norway	Secondary analysis of randomized controlled trial; Eight healthcare clinics in southern Norway	Adult women with singleton pregnancy within the first 20 weeks of gestation *n* = 606 (Intervention: 303, control: 303) ***Withdrawals*** Intervention: 8, control: 9	**Comprising dietary and physical activity components** *Physical activity components* ***Intervention group:*** ***Supervised exercise program*** Supervised group exercise sessions twice per week, each lasting for 60 min, consisting 10 min warm-up, 40 min moderate-intensity cardiovascular and strength training and 10 min stretching. ***Information dissemination*** Booklets are also provided to provide information on recommendations for healthy lifestyles. Further reinforcement of participants’ knowledge on healthy lifestyles is achieved through access to a website containing health information. ***Control group:*** Standard prenatal care	Physiotherapists and students at fitness centers	Perceived barriers to leisure-time physical activity (self-efficacy in leisure-time physical activity) Data collected at: Baseline At the end of intervention 6-month postpartum 12-month postpartum	Author-developed questionnaire	***Within-group comparisons*** Significant decrease in the number of perceived barriers to leisure-time physical activity from to 12-month postpartum for both intervention and control groups (*p* < 0.001) ***Between-group comparisons*** No significant difference in the extent of decrease in the number of these perceived barriers between groups. Significantly lower proportion of participants in the intervention group reporting certain perceived barriers, compared to control group at postintervention: ○ “Insufficient time” (being too busy): *p* = 0.03 ○ “I do not believe that I will manage” (low self-efficacy): *p* = 0.05 ○ “Fear to harm the baby”: *p* = 0.002
Hui et al. 2006; Canada	Pilot randomized controlled trial; Local community in urban Winnipeg	Pregnant women before the 26th week of pregnancy *n* = 52 (Numbers of participants randomized into the two groups are not reported) ***Withdrawals*** Intervention: Not reported, control: Not reported	**Comprising dietary and physical activity components** *Physical activity components* ***Intervention group:*** ***Supervised exercise program*** Participation in an aerobic exercise program with 3–5 exercise sessions (one at the community centers as a group session and the remaining at home) per week, each lasting 30–45 min for a period of 10–16 weeks. ***Control group:*** Standard prenatal care	Fitness instructors	Physical activity level Data collected at: Baseline Postintervention	An activity diary completed by participants	***Between-group comparison*** No significant difference in physical activity level was observed between the two groups of participants at baseline (*p* = 0.14), and intervention participants exhibited a significantly higher level of physical activity compared to controls after the intervention (*p* = 0.005)
Hui et al. 2012; Canada	Randomized controlled trial; Local community in Winnipeg	Pregnant women before the 26th week of pregnancy *n* = 224 (Intervention: 112, control: 112) ***Withdrawals*** Intervention: 10, control: 24	**Comprising dietary and physical activity components** *Physical activity components* ***Intervention group:*** ***Supervised exercise program*** Participation in an aerobic exercise program with 3–5 exercise sessions (one at the community centers as a group session and the remaining at home) per week, each lasting 30–45 min for a period of 10–16 weeks. ***Control group:*** Standard prenatal care	Fitness instructors	Physical activity level Data collected at: Baseline Two months after being enrolled	Physical activity logbooks	***Between-group comparison*** At two months after subject enrollment, the reported physical activity level among intervention participants was significantly higher than that among control participants (*p* = 0.00002)
Aşcı and Rathfisch 2016; Turkey	Randomized controlled trial; Local family health center in Istanbul	Adult pregnant women who were pregnant for less than three months *n* = 102 (Intervention: 51, control: 51) ***Withdrawals*** Intervention: 6, control: 6	**Comprising dietary and physical activity components** *Physical activity components* ***Intervention group:*** ***Counselling*** Interviews, each lasting 1 h, for educating participants on the importance on healthy lifestyle, and making recommendations on the low-level aerobic exercises, encouraging them to do moderate-intensity physical exercises regularly. ***Control group:*** Standard care	Investigator of the study	Physical activity level Data collected at: Baseline Postintervention 6 weeks at postpartum	Health Promoting Lifestyle Profile II (HPLP-II)	***Between-group comparison*** In the physical activity subscale of the HPLP-II, participants in the intervention group achieved a higher increase in the physical activity subscale score than those in the control group after the intervention (Adjusted mean difference between intervention and control groups: 3.12; 95% CI: 1.51–4.74; *p* = 0.0002)
Sklempe Kokic et al. 2017; Croatia	Secondary analysis of randomized controlled trial; Two local hospitals in Zagreb	Adult pregnant women before 30 weeks of gestation *n* = 45 (Intervention: 22, control: 23) ***Withdrawals*** Intervention: 2, control: 1	***Intervention group:*** ***Supervised exercise program*** Participation in an individual supervised exercise program, with sessions held twice per week, each lasting 50–55 min, for a duration of six week or more (throughout the participants’ pregnancy). The exercise sessions consisted of aerobic exercises on a treadmill, resistance exercises, pelvic floor exercises, stretching and relaxation. ***Unsupervised exercise program*** Participants were also asked to do brisk walking for 30 min each day ***Control group:*** Standard antenatal care	Not specified	Physical activity level Data collected at: Baseline Postintervention	Pregnancy Physical Activity Questionnaire	***Between-group comparison*** At postintervention, participants in the intervention group exhibited a significantly higher level of activity at light intensity or above (*p* = 0.027) and did a higher level of sports or exercises (*p* < 0.001) and transportation activities (*p* = 0.027), compared to control participants. Specifically, level of moderate-intensity activities among the intervention participants appeared significantly higher than that among control participants (*p* = 0.014)

## References

[1] Benefits of Regular Physical Activity. http://www.euro.who.int/en/health-topics/disease-prevention/nutrition/a-healthy-lifestyle/benefits-of-regular-physical-activity.

[2] Global Recommendations on Physical Activity for Health: 18–64 Years Old. http://www.who.int/dietphysicalactivity/physical-activity-recommendations-18-64years.pdf?ua=1.

[3] Global Recommendations on Physical Activity for Health. http://apps.who.int/iris/bitstream/handle/10665/44399/9789241599979_eng.pdf;jsessionid=712481861C37570B9F9C546472F8F3F4?sequence=1.

[4] Level of Physical Activity by WHO Recommendations. https://www.chp.gov.hk/en/statistics/data/10/280/6626.html.

[5] Guthold R., Stevens G.A., Riley L.M., Bull F.C. (2018). Worldwide trends in insufficient physical activity from 2001 to 2016: A pooled analysis of 358 population-based surveys with 1·9 million participants. Lancet Glob. Health.

[6] Downs D.S., Chasan-Taber L., Evenson K.R., Leiferman J., Yeo S. (2012). Physical activity and pregnancy: Past and present evidence and future recommendations. Res. Q. Exerc. Sport.

[7] Ruifrok A.E., Althuizen E., Oostdam N., van Mechelen W., Mol B.W., de Groot C.J., van Poppel M.N. (2014). The relationship of objectively measured physical activity and sedentary behaviour with gestational weight gain and birth weight. J. Pregnancy.

[8] Padmapriya N., Shen L., Soh S.E., Shen Z., Kwek K., Godfrey K.M., Gluckman P.D., Chong Y.S., Saw S.M., Müller-Riemenschneider F. (2015). Physical activity and sedentary behavior patterns before and during pregnancy in a multi-ethnic sample of Asian women in Singapore. Matern. Child Health J..

[9] Gaston A., Cramp A. (2011). Exercise during pregnancy: A review of patterns and determinants. J. Sci. Med. Sport.

[10] Fazzi C., Saunders D.H., Linton K., Norman J.E., Reynolds R.M. (2017). Sedentary behaviours during pregnancy: A systematic review. Int. J. Behav. Nutr. Phys. Act..

[11] Evenson K.R., Savitz D.A., Huston S.L. (2004). Leisure-time physical activity among pregnant women in the US. Paediatr. Perinat. Epidemiol..

[12] Löf M. (2011). Physical activity pattern and activity energy expenditure in healthy pregnant and non-pregnant Swedish women. Eur. J. Clin. Nutr..

[13] Wu W.H., Meijer O.G., Uegaki K., Mens J.M., van Dieën J.H., Wuisman P.I., Ostgaard H.C. (2004). Pregnancy-related pelvic girdle pain (PPP), I: Terminology, clinical presentation, and prevalence. Eur. Spine J..

[14] Littleton H.L., Breitkopf C.R., Berenson A.B. (2007). Correlates of anxiety symptoms during pregnancy and association with perinatal outcomes: A meta-analysis. Am. J. Obstet. Gynecol..

[15] Kominiarek M.A., Peaceman A.M. (2017). Gestational weight gain. Am. J. Obstet. Gynecol..

[16] Lardon E., St-Laurent A., Babineau V., Descarreaux M., Ruchat S.M. (2018). Lumbopelvic pain, anxiety, physical activity and mode of conception: A prospective cohort study of pregnant women. BMJ Open.

[17] De Wit L., Jelsma J.G., van Poppel M.N., Bogaerts A., Simmons D., Desoye G., Corcoy R., Kautzky-Willer A., Harreiter J., van Assche A. (2015). Physical activity, depressed mood and pregnancy worries in European obese pregnant women: Results from the DALI study. BMC Pregnancy Childbirth.

[18] Evenson K.R., Moos M.K., Carrier K., Siega-Riz A.M. (2009). Perceived barriers to physical activity among pregnant women. Matern. Child Health J..

[19] Zhang Y., Dong S., Zuo J., Hu X., Zhang H., Zhao Y. (2014). Physical activity level of urban pregnant women in Tianjin, China: A cross-sectional study. PLoS ONE.

[20] Lee D.T., Ngai I.S., Ng M.M., Lok I.H., Yip A.S., Chung T.K. (2009). Antenatal taboos among Chinese women in Hong Kong. Midwifery.

[21] Clarke P.E., Rousham E.K., Gross H., Halligan A.W., Bosio P. (2005). Activity patterns and time allocation during pregnancy: A longitudinal study of British women. Ann. Hum. Biol..

[22] Fell D.B., Joseph K.S., Armson B.A., Dodds L. (2009). The impact of pregnancy on physical activity level. Matern. Child Health J..

[23] Ning Y., Williams M.A., Dempsey J.C., Sorensen T.K., Frederick I.O., Luthy D.A. (2003). Correlates of recreational physical activity in early pregnancy. J. Matern. Fetal Neonatal Med..

[24] Pereira M.A., Rifas-Shiman S.L., Kleinman K.P., Rich-Edwards J.W., Peterson K.E., Gillman M.W. (2007). Predictors of change in physical activity during and after pregnancy: Project viva. Am. J. Prev. Med..

[25] Schmidt M.D., Pekow P., Freedson P.S., Markenson G., Chasan-Taber L. (2006). Physical activity patterns during pregnancy in a diverse population of women. J. Women’s Health (Larchmt).

[26] Phelan S. (2010). Pregnancy: A “teachable moment” for weight control and obesity prevention. Am. J. Obstet. Gynecol..

[27] Streuling I., Beyerlein A., Rosenfeld E., Hofmann H., Schulz T., von Kries R. (2011). Physical activity and gestational weight gain: A meta-analysis of intervention trials. BJOG.

[28] Dempsey J.C., Butler C.L., Sorensen T.K., Lee I.M., Thompson M.L., Miller R.S., Frederick I.O., Williams M.A. (2004). A case-control study of maternal recreational physical activity and risk of gestational diabetes mellitus. Diabetes Res. Clin. Pract..

[29] Mørkrid K., Jenum A.K., Berntsen S., Sletner L., Richardsen K.R., Vangen S., Holme I., Birkeland K.I. (2014). Objectively recorded physical activity and the association with gestational diabetes. Scand. J. Med. Sci. Sports.

[30] Han S., Middleton P., Crowther C.A. (2012). Exercise for pregnant women for preventing gestational diabetes mellitus. Cochrane Database Syst. Rev..

[31] Van Poppel M.N., Ruchat S.M., Mottola M.F. (2014). Physical activity and gestational diabetes mellitus. Med. Sport Sci..

[32] Aune D., Saugstad O.D., Henriksen T., Tonstad S. (2014). Physical activity and the risk of preeclampsia: A systematic review and meta-analysis. Epidemiology.

[33] Kasawara K.T., do Nascimento S.L., Costa M.L., Surita F.G., e Silva J.L. (2012). Exercise and physical activity in the prevention of pre-eclampsia: Systematic review. Acta Obstet. Gynecol. Scand..

[34] Davenport M.H., Marchand A.A., Mottola M.F., Poitras V.J., Gray C.E., Jaramillo Garcia A., Barrowman N., Sobierajski F., James M., Meah V.L. (2019). Exercise for the prevention and treatment of low back, pelvic girdle and lumbopelvic pain during pregnancy: A systematic review and meta-analysis. Br. J. Sports Med..

[35] Hinckley A.F., Bachand A.M., Reif J.S. (2005). Late pregnancy exposures to disinfection by-products and growth-related birth outcomes. Environ. Health Perspect..

[36] Barakat R., Pelaez M., Montejo R., Luaces M., Zakynthinaki M. (2011). Exercise during pregnancy improves maternal health perception: A randomized controlled trial. Am. J. Obstet. Gynecol..

[37] Gaston A., Prapavessis H. (2013). Tired, moody and pregnant? Exercise may be the answer. Psychol. Health.

[38] Loprinzi P.D., Fitzgerald E.M., Cardinal B.J. (2012). Physical activity and depression symptoms among pregnant women from the National Health and Nutrition Examination Survey 2005–2006. J. Obstet. Gynecol. Neonatal Nurs..

[39] Perales M., Refoyo I., Coteron J., Bacchi M., Barakat R. (2015). Exercise during pregnancy attenuates prenatal depression: A randomized controlled trial. Eval. Health Prof..

[40] Barakat R., Pelaez M., Montejo R., Refoyo I., Coteron J. (2014). Exercise throughout pregnancy does not cause preterm delivery: A randomized, controlled trial. J. Phys. Act. Health.

[41] Haakstad L.A., Bø K. (2011). Exercise in pregnant women and birth weight: A randomized controlled trial. BMC Pregnancy Childbirth.

[42] Evenson K.R., Barakat R., Brown W.J., Dargent-Molina P., Haruna M., Mikkelsen E.M., Mottola M.F., Owe K.M., Rousham E.K., Yeo S. (2014). Guidelines for Physical Activity during Pregnancy: Comparisons From Around the World. Am. J. Lifestyle Med..

[43] Harrison C.L., Lombard C.B., Teede H.J. (2014). Limiting postpartum weight retention through early antenatal intervention: The HeLP-her randomised controlled trial. Int. J. Behav. Nutr. Phys. Act..

[44] Hawkins M., Chasan-Taber L., Marcus B., Stanek E., Braun B., Ciccolo J., Markenson G. (2014). Impact of an exercise intervention on physical activity during pregnancy: The behaviors affecting baby and you study. Am. J. Public Health.

[45] Lewis B.A., Martinson B.C., Sherwood N.E., Avery M.D. (2011). A pilot study evaluating a telephone-based exercise intervention for pregnant and postpartum women. J. Midwifery Women’s Health.

[46] Gaston A., Prapavessis H. (2009). Maternal-fetal disease information as a source of exercise motivation during pregnancy. Health Psychol..

[47] Currie S., Sinclair M., Murphy M.H., Madden E., Dunwoody L., Liddle D. (2013). Reducing the decline in physical activity during pregnancy: A systematic review of behavior change interventions. PLoS ONE.

[48] Huang T.T., Yeh C.Y., Tsai Y.C. (2011). A diet and physical activity intervention for preventing weight retention among Taiwanese childbearing women: A randomised controlled trial. Midwifery.

[49] Luoto R., Kinnunen T.I., Aittasalo M., Kolu P., Raitanen J., Ojala K., Mansikkamäki K., Lamberg S., Vasankari T., Komulainen T. (2011). Primary prevention of gestational diabetes mellitus and large-for-gestational-age newborns by lifestyle counseling: A cluster-randomized controlled trial. PLoS Med..

[50] Callaway L.K., Colditz P.B., Byrne N.M., Lingwood B.E., Rowlands I.J., Foxcroft K., McIntyre H.D., BAMBINO Group (2010). Prevention of gestational diabetes: Feasibility issues for an exercise intervention in obese pregnant women. Diabetes Care.

[51] Guelinckx I., Devlieger R., Mullie P., Vansant G. (2010). Effect of lifestyle intervention on dietary habits, physical activity, and gestational weight gain in obese pregnant women: A randomized controlled trial. Am. J. Clin. Nutr..

[52] Hui A., Ludwig S., Gardiner P., Sevenhuysen G., Murray R., Morris M., Shen G.X. (2006). Community-based exercise and dietary intervention during pregnancy: A pilot study. Can. J. Diabetes.

[53] Grunebaum A., Dudenhausen J.W., Ross C.M. (2015). Compliance with the institute of medicine gestational weight gain recommendations in teenage pregnancies. Obstet. Gynecol..

[54] Evans N., Lasen M., Tsey K. (2015). A Systematic Review of Rural Development Research.

[55] Picot J., Hartwell D., Harris P., Mendes D., Clegg A.J., Takeda A. (2012). The effectiveness of interventions to treat severe acute malnutrition in young children: A systematic review. Health Technol. Assess..

[56] Stafne S.N., Salvesen K.A., Romundstad P.R., Stuge B., Mørkved S. (2012). Does regular exercise during pregnancy influence lumbopelvic pain? A randomized controlled trial. Acta Obstet. Gynecol. Scand..

[57] Songoygard K.M., Stafne S.N., Evensen K.A., Salvesen K.A., Vik T., Morkved S. (2012). Does exercise during pregnancy prevent postnatal depression? A randomized controlled trial. Acta Obstet. Gynecol. Scand..

[58] Gustafsson M.K., Stafne S.N., Romundstad P.R., Mørkved S., Salvesen K., Helvik A.S. (2016). The effects of an exercise program during pregnancy on health-related quality of life in pregnant women: A Norwegian randomised controlled trial. BJOG.

[59] Eggen M.H., Stuge B., Mowinckel P., Jensen K.S., Hagen K.B. (2012). Can supervised group exercises including ergonomic advice reduce the prevalence and severity of low back pain and pelvic girdle pain in pregnancy? A randomized controlled trial. Phys. Ther..

[60] Sagedal L.R., Øverby N.C., Bere E., Torstveit M.K., Lohne-Seiler H., Småstuen M., Hillesund E.R., Henriksen T., Vistad I. (2017). Lifestyle intervention to limit gestational weight gain: The Norwegian Fit for Delivery randomised controlled trial. BJOG.

[61] Haakstad L.A., Vistad I., Sagedal L.R., Lohne-Seiler H., Torstveit M.K. (2018). How does a lifestyle intervention during pregnancy influence perceived barriers to leisure-time physical activity? The Norwegian fit for delivery study, a randomized controlled trial. BMC Pregnancy Childbirth.

[62] Haakstad L.A., Bø K. (2011). Effect of regular exercise on prevention of excessive weight gain in pregnancy: A randomised controlled trial. Eur. J. Contracept. Reprod. Health Care.

[63] Haakstad L.A., Bø K. (2015). Effect of a regular exercise program on pelvic girdle and low back pain in previously inactive pregnant women: A randomized controlled trial. J. Rehabil. Med..

[64] Haakstad L.A., Torset B., Bø K. (2016). What is the effect of regular group exercise on maternal psychological outcomes and common pregnancy complaints? An assessor blinded RCT. Midwifery.

[65] Ozdemir S., Bebis H., Ortabag T., Acikel C. (2015). Evaluation of the efficacy of an exercise program for pregnant women with low back and pelvic pain: A prospective randomized controlled trial. J. Adv. Nurs..

[66] Aşcı O., Rathfisch G. (2016). Effect of lifestyle interventions of pregnant women on their dietary habits, lifestyle behaviors, and weight gain: A randomized controlled trial. J. Health Popul. Nutr..

[67] Ruiz J.R., Perales M., Pelaez M., Lopez C., Lucia A., Barakat R. (2013). Supervised exercise-based intervention to prevent excessive gestational weight gain: A randomized controlled trial. Mayo Clin. Proc..

[68] Kokic I.S., Ivanisevic M., Uremovic M., Kokic T., Pisot R., Simunic B. (2017). Effect of therapeutic exercises on pregnancy-related low back pain and pelvic girdle pain: Secondary analysis of a randomized controlled trial. J. Rehabil. Med..

[69] Kinnunen T.I., Pasanen M., Aittasalo M., Fogelholm M., Hilakivi-Clarke L., Weiderpass E., Luoto R. (2007). Preventing excessive weight gain during pregnancy—A controlled trial in primary health care. Eur. J. Clin. Nutr..

[70] Ronnberg A.K., Ostlund I., Fadl H., Gottvall T., Nilsson K. (2015). Intervention during pregnancy to reduce excessive gestational weight gain—A randomised controlled trial. BJOG.

[71] Miquelutti M.A., Cecatti J.G., Makuch M.Y. (2013). Evaluation of a birth preparation program on lumbopelvic pain, urinary incontinence, anxiety and exercise: A randomized controlled trial. BMC Pregnancy Childbirth.

[72] Da Silva S.G., Hallal P.C., Domingues M.R., Bertoldi A.D., Silveira M.F.D., Bassani D., da Silva I.C.M., da Silva B.G.C., Coll C.V.N., Evenson K. (2017). A randomized controlled trial of exercise during pregnancy on maternal and neonatal outcomes: Results from the PAMELA study. Int. J. Behav. Nutr. Phys. Act..

[73] Hui A., Back L., Ludwig S., Gardiner P., Sevenhuysen G., Dean H., Sellers E., McGavock J., Morris M., Bruce S. (2012). Lifestyle intervention on diet and exercise reduced excessive gestational weight gain in pregnant women under a randomised controlled trial. BJOG.

[74] Arizabaleta A.V.M., Buitrago L.O., de Plata A.C.A., Escudero M.M., Ramirez-Velez R. (2010). Aerobic exercise during pregnancy improves health-related quality of life: A randomised trial. J. Physiother..

[75] Robledo-Colonia A.F., Sandoval-Restrepo N., Mosquera-Valderrama Y.F., Escobar-Hurtado C., Ramírez-Vélez R. (2012). Aerobic exercise training during pregnancy reduces depressive symptoms in nulliparous women: A randomised trial. J. Physiother..

[76] Marquez-Sterling S., Perry A.C., Kaplan T.A., Halberstein R.A., Signorile J.F. (2000). Physical and psychological changes with vigorous exercise in sedentary primigravidae. Med. Sci. Sports Exerc..

[77] Garshasbi A., Faghih Zadeh S. (2005). The effect of exercise on the intensity of low back pain in pregnant women. Int. J. Gynaecol. Obstet..

[78] Ghodsi Z., Asltoghiri M. (2014). Effects of aerobic exercise training on maternal and neonatal outcome: A randomized controlled trial on pregnant women in Iran. J. Pak. Med. Assoc..

[79] Gau M.L., Chang C.Y., Tian S.H., Lin K.C. (2011). Effects of birth ball exercise on pain and self-efficacy during childbirth: A randomised controlled trial in Taiwan. Midwifery.

[80] Kluge J., Hall D., Louw Q., Theron G., Grové D. (2011). Specific exercises to treat pregnancy-related low back pain in a South African population. Int. J. Gynaecol. Obstet..

[81] Suputtitada A., Wacharapreechanont T., Chaisayan P. (2002). Effect of the “sitting pelvic tilt exercise” during the third trimester in primigravidas on back pain. J. Med. Assoc. Thail..

[82] Khodaveisi M., Omidi A., Farokhi S., Soltanian A.R. (2017). The effect of pender’s health promotion model in improving the nutritional behavior of overweight and obese women. Int. J. Community Based Nurs. Midwifery.

[83] Laitakari J., Asikainen T.M. (1998). How to promote physical activity through individual counseling—A proposal for a practical model of counseling on health-related physical activity. Patient Educ. Couns..

[84] Physical Activity and Exercise during Pregnancy and the Postpartum Period. https://www.acog.org/Clinical-Guidance-and-Publications/Committee-Opinions/Committee-on-Obstetric-Practice/Physical-Activity-and-Exercise-During-Pregnancy-and-the-Postpartum-Period.

[85] Roomruangwong C., Kanchanatawan B., Sirivichayakul S., Maes M. (2017). High incidence of body image dissatisfaction in pregnancy and the postnatal period: Associations with depression, anxiety, body mass index and weight gain during pregnancy. Sex. Reprod. Healthc..

[86] Price B.B., Amini S.B., Kappeler K. (2012). Exercise in pregnancy: Effect on fitness and obstetric outcomes-a randomized trial. Med. Sci. Sports Exerc..

[87] Cid M., González M. (2016). Potential benefits of physical activity during pregnancy for the reduction of gestational diabetes prevalence and oxidative stress. Early Hum. Dev..

[88] Padmapriya N., Bernard J.Y., Liang S., Loy S.L., Shen Z., Kwek K., Godfrey K.M., Gluckman P.D., Chong Y.S., Saw S.M. (2016). Association of physical activity and sedentary behavior with depression and anxiety symptoms during pregnancy in a multiethnic cohort of Asian women. Arch. Womens Ment. Health.

[89] Shakeel N., Richardsen K.R., Martinsen E.W., Eberhard-Gran M., Slinning K., Jenum A.K. (2018). Physical activity in pregnancy and postpartum depressive symptoms in a multiethnic cohort. J. Affect. Disord..

[90] Goodwin A., Astbury J., McMeeken J. (2000). Body image and psychological well-being in pregnancy. A comparison of exercisers and non-exercisers. Aust. N. Z. J. Obstet. Gynaecol..

[91] Bisson M., Alméras N., Dufresne S.S., Robitaille J., Rhéaume C., Bujold E., Frenette J., Tremblay A., Marc I. (2015). A 12-week exercise program for pregnant women with obesity to improve physical activity levels: An open randomised preliminary study. PLoS ONE.

[92] Engberg E., Tikkanen H.O., Koponen A., Hägglund H., Kukkonen-Harjula K., Tiitinen A., Peltonen J.E., Pöyhönen-Alho M. (2018). Cardiorespiratory fitness and health-related quality of life in women at risk for gestational diabetes. Scand. J. Med. Sci. Sports.

[93] Chan D.N., So W.K. (2015). A systematic review of randomised controlled trials examining the effectiveness of breast and cervical cancer screening interventions for ethnic minority women. Eur. J. Oncol. Nurs..

